# Projected outcomes of universal testing and treatment in a generalised HIV epidemic in Zambia and South Africa (the HPTN 071 [PopART] trial): a modelling study

**DOI:** 10.1016/S2352-3018(22)00259-4

**Published:** 2022-11-01

**Authors:** William J M Probert, Rafael Sauter, Michael Pickles, Anne Cori, Nomtha F Bell-Mandla, Justin Bwalya, Lucie Abeler-Dörner, Peter Bock, Deborah J Donnell, Sian Floyd, David Macleod, Estelle Piwowar-Manning, Timothy Skalland, Kwame Shanaube, Ethan Wilson, Blia Yang, Helen Ayles, Sarah Fidler, Richard J Hayes, Christophe Fraser, HPTN 071 (PopART) Study Team, Richard Hayes, Sarah Fidler, Nulda Beyers, Helen Ayles, Peter Bock, Wafaa El-Sadr, Myron Cohen, Susan Eshleman, Yaw Agyei, Estelle Piwowar-Manning, Virginia Bond, Graeme Hoddinott, Deborah Donnell, Sian Floyd, Ethan Wilson, Lynda Emel, Heather Noble, David Macleod, David Burns, Christophe Fraser, Anne Cori, Nirupama Sista, Sam Griffith, Ayana Moore, Tanette Headen, Rhonda White, Eric Miller, James Hargreaves, Katharina Hauck, Ranjeeta Thomas, Mohammed Limbada, Justin Bwalya, Michael Pickles, Kalpana Sabapathy, Ab Schaap, Rory Dunbar, Kwame Shanaube, Blia Yang, Musonda Simwinga, Peter Smith, Sten Vermund, Nomtha Mandla, Nozizwe Makola, Anneen van Deventer, Anelet James, Karen Jennings, James Kruger, Mwelwa Phiri, Barry Kosloff, Lawrence Mwenge, Sarah Kanema, Rafael Sauter, William Probert, Ramya Kumar, Ephraim Sakala, Andrew Silumesi, Tim Skalland, Krista Yuhas

**Affiliations:** a Big Data Institute, Li Ka Shing Centre for Health Information and Discovery, University of Oxford, Oxford, UK; b Medical Research Council Centre for Global Infectious Disease Analysis, School of Public Health, Imperial College London, London, UK; c Department of Infectious Disease, Imperial College London, London, UK; d Desmond Tutu TB Centre, Department of Paediatrics and Child Health, Faculty of Medicine and Health Sciences, Stellenbosch University, Cape Town, South Africa; e Zambart, University of Zambia, Lusaka, Zambia; f Fred Hutchinson Cancer Research Center, Seattle, WA, USA; g Faculty of Epidemiology and Population Health, London School of Hygiene & Tropical Medicine, London, UK; h School of Medicine, Johns Hopkins University, Baltimore, MD, USA; i NIHR Imperial Biomedical Research Centre, London, UK

## Abstract

**Background:**

The long-term impact of universal home-based testing and treatment as part of universal testing and treatment (UTT) on HIV incidence is unknown. We made projections using a detailed individual-based model of the effect of the intervention delivered in the HPTN 071 (PopART) cluster-randomised trial.

**Methods:**

In this modelling study, we fitted an individual-based model to the HIV epidemic and HIV care cascade in 21 high prevalence communities in Zambia and South Africa that were part of the PopART cluster-randomised trial (intervention period Nov 1, 2013, to Dec 31, 2017). The model represents coverage of home-based testing and counselling by age and sex, delivered as part of the trial, antiretroviral therapy (ART) uptake, and any changes in national guidelines on ART eligibility. In PopART, communities were randomly assigned to one of three arms: arm A received the full PopART intervention for all individuals who tested positive for HIV, arm B received the intervention with ART provided in accordance with national guidelines, and arm C received standard of care. We fitted the model to trial data twice using Approximate Bayesian Computation, once before data unblinding and then again after data unblinding. We compared projections of intervention impact with observed effects, and for four different scenarios of UTT up to Jan 1, 2030 in the study communities.

**Findings:**

Compared with standard of care, a 51% (95% credible interval 40–60) reduction in HIV incidence is projected if the trial intervention (arms A and B combined) is continued from 2020 to 2030, over and above a declining trend in HIV incidence under standard of care.

**Interpretation:**

A widespread and continued commitment to UTT via home-based testing and counselling can have a substantial effect on HIV incidence in high prevalence communities.

**Funding:**

National Institute of Allergy and Infectious Diseases, US President's Emergency Plan for AIDS Relief, International Initiative for Impact Evaluation, Bill & Melinda Gates Foundation, National Institute on Drug Abuse, and National Institute of Mental Health.

## Introduction

Much progress has been made in HIV prevention since the widespread roll-out of HIV testing and treatment and voluntary medical male circumcision (VMMC) in combination prevention packages.^1^ Despite globally decreasing incidences of HIV, in 2020 the number of new HIV infections reached 1·5 million worldwide, well above the UNAIDS Fast-Track target of less than 500 000 new HIV infections per year by 2020.^2^

Universal testing and treatment (UTT) has been proposed as a method to substantially curb growth of the global HIV epidemic.^3^, ^4^ Four trials assessing the effect of UTT on community HIV incidence have reported mixed results:^5^, ^6^ two reported a non-significant effect despite observed decreases in HIV incidence,^7^, ^8^, ^9^ and two reported reductions in community HIV incidence of 20–30% over 3 years.^10^, ^11^

Several reasons have been proposed for these varying reported effects of UTT.^6^, ^10^ There remains a need to understand and quantify the effects of UTT beyond the relatively short study periods of cluster-randomised trials within communities with high HIV prevalence.

HPTN 071 (PopART) was a cluster-randomised trial conducted in 21 high prevalence communities in South Africa and Zambia with the intervention occurring between Nov 1, 2013, and Dec 31, 2017.^12^ The trial intervention (hereafter referred to as the PopART intervention) comprised annual rounds of home-based HIV testing, including support for linkage to care and retention on antiretroviral therapy (ART) at government primary health-care facilities, promotion of VMMC for HIV-negative men, and additional HIV, tuberculosis, and sexually transmitted infection services, and was delivered by teams of community HIV-care providers. Communities comprised seven triplets, matched on HIV prevalence and geographical location. Within each triplet, communities were randomly allocated into one of three arms: arm A received the full combination HIV prevention package with the offer of immediate ART for individuals testing positive for HIV; arm B received the full combination HIV prevention package with ART initiation following national guidelines; and arm C received standard of care. From late 2016, ART for all HIV-positive individuals was standard of care in both countries, aligning arms A and B. The primary outcome of the trial was the reduction in HIV incidence between arms A or B and C between months 12 and 36, as measured in a population cohort of approximately 2000 randomly sampled adults (age 18–44 years) from each community, who were followed up annually (appendix p 5). Within the trial, the relative reduction in HIV incidence was 7% comparing arm A to arm C (adjusted rate ratio: 0·93 [95% CI 0·74–1·18]), 30% comparing arm B to arm C (0·70 [0·55–0·88]), and 19% in a combined analysis of arms A and B versus arm C (0·81 [0·66–0·99]).^10^

**Research in context**

**Evidence before this study**

The effect of expanded access to HIV care on HIV incidence has been investigated in three areas: modelling studies, cluster-randomised trials, and retrospective analyses. To date, there have been four cluster-randomised trials looking at the effect of universal testing and treatment (UTT) on HIV incidence at the population level: two reported no effect on HIV incidence and two reported 20–30% reduction in HIV incidence over a 3-year period. Several modelling studies have looked at the effect of UTT on HIV incidence. Since 2017, one other UTT trial has also reported modelling results over a 3-year period (from mid-2013 to mid-2016), and observed a 5% relative reduction in incidence, projecting 4–40% reduction in incidence before data unblinding, 43% after data unblinding.

**Added value of this study**

In this study, we incorporated mathematical modelling since the start of a cluster-randomised trial of an HIV prevention intervention. This is the largest HIV prevention trial to date. This study makes projections over the trial period (2013–17) and extrapolates effect up to 2030. The modelling team followed a transparent analysis process by preregistering a modelling analysis plan and closely followed the preregistered statistical analysis plan. The modelling team, which was blinded to data reporting of the primary outcome of the trial, made projections of the effect of the intervention before and after data unblinding, thereby providing a framework to test the predictive efficacy of the model.

**Implications of all the available evidence**

A widespread and continued commitment to UTT via home-based testing and counselling could have a substantial effect on HIV incidence in high prevalence communities.

PopART incorporated a modelling component from inception^12^ and throughout the trial, including pre-trial projections of the effect of the intervention,^13^ providing three reports of projections to the independent data safety and monitoring board (DSMB; appendix p 28), contributing to the cost-effectiveness analysis of the intervention,^14^ and comparisons with phylogenetic analyses.^15^

The data unblinding of the primary endpoint of the trial on Dec 17, 2018,^16^ provided an opportunity to test model predictions. The modelling team preregistered the intended analyses surrounding the data unblinding.^17^

In this study, we aimed to answer three questions. What is the predictive ability of the PopART individual-based model with respect to the primary outcome of the HPTN 071 (PopART) trial, reduction in HIV incidence between arms A or B and C, and selected secondary endpoints? What is the reason for the mismatches, if any, between the projected and realised trial outcome according to the model? And, after resolving these mismatches, if any, what are the projected long-term effects of a sustained PopART intervention, as implemented in the trial?

## Methods

### Study design, model, and inference framework

In this modelling study, we used the PopART individual-based model, a stochastic simulation model of the HIV epidemic in the 21 communities of the trial.^18^, ^19^ Each PopART community was simulated as a system of two geographical areas referred to as patches, in which one patch receives the PopART intervention (if it is an intervention community) and the other patch represents the surrounding area, accounting for partnerships forming between individuals in the PopART community and the surrounding area. The model introduces HIV during 1970–80, transmission is modelled in serodiscordant heterosexual partnerships, and HIV disease progression in the absence of ART is based on data from the ATHENA cohort.^20^

Both the PopART intervention and standard of care were modelled, and both include HIV testing, ART, VMMC, and changes in national guidelines regarding eligibility for ART. The simulated intervention matches the observed age-stratified and sex-stratified proportion of individuals successfully visited by a community HIV-care providers team each week. Individuals adherent to ART are assumed to not transmit HIV, and treatment failure and poor ART adherence are modelled via the proportion of individuals receiving treatment becoming virally unsuppressed. Treatment dropout is modelled with rates reported in the HIV literature.^18^

Inference was done independently for each community. Parameters were chosen to be estimated within the calibration process if they were expected to vary across communities, could be estimated from data, or represented quantities about which there was considerable uncertainty (and thereby included to represent uncertainty adequately). Several model parameters were estimated by pairing the model with Approximate Bayesian Computation.^21^ Projections are based on 1000 accepted parameters, with results reported as the median and 95% credible interval (CrI; 2·5% and 97·5% quantiles). All model parameters and details of the model and inference framework have been provided elsewhere.^18^

### Data sources

We used demographic data on mortality and fertility from UN databases for 1950–2050. We used data from the population cohort to inform simulation of three groups of sexual risk-taking behaviour, partnership duration for each of these groups, and prevalence of traditional male circumcision in each community. We used data from the PopART intervention (ie, community HIV-care provider data) to determine testing coverage of the intervention.

**Figure 1:**
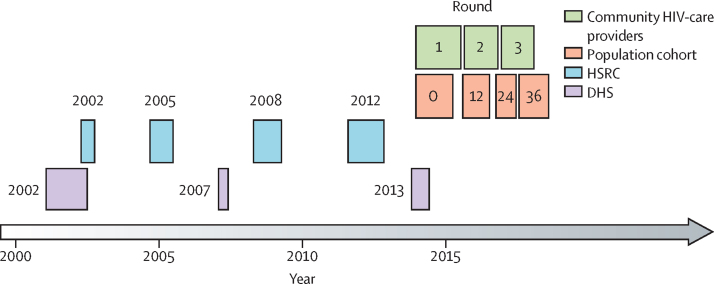
Timing of different data sources used in model fitting Demographic and Health Surveys (DHS) were used in Zambian communities, surveys from the Human Sciences Research Council (HSRC) were used in South Africa.

We used four types of data for model fitting, each stratified by age and sex: HIV prevalence, proportion aware of HIV status among people with HIV, self-reported proportion on ART among those aware of their positive HIV status, and the proportion of individuals who are virally suppressed among people with HIV. These data were from four cohorts over different time periods (figure 1): four rounds from the population cohort (collected during the trial in 2014–18; age 18–44 years), three rounds of community HIV-care provider data (collected during the trial in 2013–17; age 18–80 years), three pre-trial rounds of the Demographic and Health Surveys in Zambia (2002, 2007, and 2013; age 15–59 years), and four pre-trial rounds of Human Sciences Research Council surveys in South Africa (2002, 2005, 2008, and 2012; age groups 15–59 years, and ≥60 years). South Africa only provided community HIV-care provider data from round 3.

Timepoints and periods within the trial are referenced in relation to sampling rounds of the population cohort: population cohort 0, population cohort 12, population cohort 24, and population cohort 36 refer to the number of months after trial start (eg, population cohort 0 is trial baseline, and population cohort 12–36 refers to the period of time between 12 and 36 months after the start of the trial).

### Data unblinding process

Model projections were made available for external review at several timepoints before data unblinding (appendix p 29): before collection of detailed trial data using a deterministic compartmental model (different from the model used here),^13^ reports using the model in this research were delivered to an independent DSMB throughout the trial; and immediately before data unblinding (for all intervention communities; henceforth called pre-unblinding projections). Projections were also assessed after data unblinding (for all communities of the trial including arm C; henceforth called post-unblinding projections; appendix p 28). Modelling analyses before and after data unblinding were prespecified.^17^

Before data unblinding for the primary endpoint of the trial, because neither community HIV-care provider nor post-baseline population cohort data were available to calibrate the model to arm C communities, we did our calculations of intervention effect using comparisons with counterfactual simulations in which the model is rerun with the same parameters except the intervention is turned off; thus, it has the same epidemic history until the start of the trial, but then continues without the simulated PopART intervention. We define this assessment as a paired counterfactual.

After unblinding, data on viral suppression at population cohort 24 were available. To improve the post-unblinding model fit to the proportion of individuals aware of their HIV status among people with HIV, the proportion of individuals on ART among those aware of status, and the proportion of individuals virally suppressed among people with HIV, we included a sex-specific multiplier in the inference framework on both the annual probability of an HIV test with standard of care and on the probability of becoming virally suppressed after linkage to care. We updated parameters governing sexual partnership formation and dissolution using the population cohort data from all four rounds. The data we used before and after data unblinding are shown in the appendix (pp 28–32).

### Modelled scenarios to 2030 and outcomes

Using the model after data unblinding, we simulated four scenarios, with model simulations running from before Jan 1, 1990, up to Jan 1, 2030. In scenario 1, we simulated the PopART intervention and continuation of the PopART intervention after the end of the trial (Dec 31, 2017) in each PopART community, but with standard of care and no PopART intervention in the surrounding area. In scenario 2, we simulated the PopART intervention and introduction of standard of care after the end of the trial (ie, discontinuation of the PopART intervention after Dec 31, 2017) and standard of care and no PopART intervention in the surrounding area. In scenario 3, we simulated no PopART intervention; instead we simulated standard of care everywhere until Jan 1, 2020, and then roll-out of the PopART intervention everywhere, akin to a nationwide roll-out of community HIV-care providers. In scenario 4, we simulated standard of care everywhere (this was a counterfactual scenario).

The primary endpoint in this analysis was the relative reduction in HIV incidence in the population cohort and in the whole population. For pre-unblinding projections, the reduction was compared with counterfactual projections; for post-unblinding projections, the reduction was compared with arm C projections.

Secondary endpoints were HIV-related mortality, the proportion of HIV incidence attributable to the acute and early phase of HIV infection, VMMC, prevalence of detectable viraemia, reproduction number (R), loss to follow-up or dropout, and treatment failure.

For observed data from the HPTN 071 (PopART) trial, estimates are presented with 95% CIs, whereas for projections made, estimates are presented with 95% CrIs.

The PopART individual-based model is written in C. We did all statistical analyses, data processing, and visualisation in Python (version 3.8) and R (version 3.6.3).

### Role of the funding source

The funders of the study had no role in data collection, data analysis, data interpretation, writing of the report, or the decision to submit for publication.

## Results

**Figure 2:**
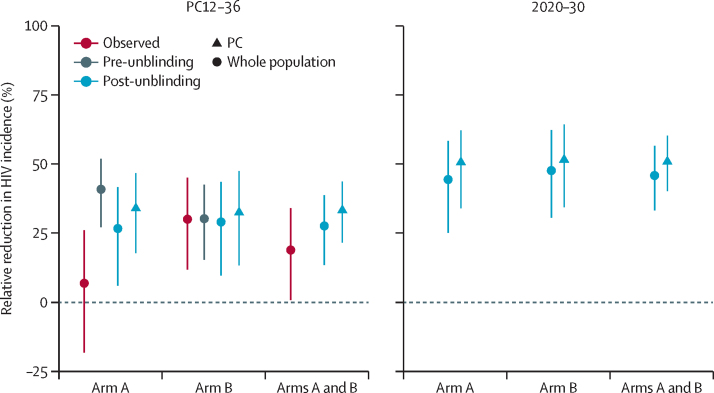
Predicted intervention impact, measured as relative reduction in HIV incidence, projected over a 12–36-month period and from 2020 to 2030 Data are geometric mean reductions in incidence, with 95% credible intervals taken across all communities and across 1000 parameter sets from the calibration framework. Projections are made for the PC age range (18–44 years) and the whole population for the period of 12–36 months after the start of the trial (PC12–36) with both the pre-unblinding model and the post-unblinding model. Projections for 2020–30 are under a continuation of the PopART intervention to 2030 in the trial community (scenario 1). Observed estimates of intervention effect from the statistical analysis of trial data are provided for the PC12–36 period with 95% CIs. The pre-unblinding model projections compare intervention communities to counterfactual simulations of the same intervention communities; all other projections compare intervention communities to their matched arm C community in the same triplet (and 95% credible intervals of model output). PC=population cohort

Prediction intervals from the model of intervention effect included the point estimate of the intervention effect from observed trial data before data unblinding in arm B comparisons with counterfactual simulations but not in arm A comparisons, and after data unblinding in comparisons of arm C with both intervention arms (figure 2). Using the post-unblinding model, the predicted relative reduction in HIV incidence over population cohort 12–36 was 27% (95% CrI 6 to 42) for arm A versus the observed 7% (95% CI –18 to 26), and 29% (95% CrI 10 to 43) in arm B versus the observed 30% (95% CI 12 to 45; figure 2). The observed effect in arm A lies in the 3rd percentile of simulated results. Before data unblinding, the effect was projected to be 41% (95% CrI 27 to 52) for arm A and 30% (16 to 42) for arm B (figure 2). All model projections mentioned hereafter refer to those generated from the post-unblinding model, unless otherwise specified.

For the whole population, the effect of the intervention compared with standard of care was predicted to be 5–7 percentage points higher over population cohort 12–36 on average than in the population cohort: the model predicted a reduction of 34% (95% CrI 18–47) in arm A and 32% (13–47) in arm B over the same period in this population (figure 2). This difference arises because the intervention had a larger effect in older age groups because of increased ART coverage and viral suppression compared with younger age groups^10^ and in men due to higher ART coverage and viral suppression than in women (the population cohort only included people aged 18–44 years and comprised approximately 70% women, whereas the whole population had a roughly 50:50 sex ratio and includes people aged 13–80 years). Despite a smaller mean absolute decrease in HIV incidence, we predicted the intervention effect to be larger in men than in women (appendix p 6). We did post-hoc analyses on arms A and B combined, motivated by alignment of these arms after universal ART was introduced in 2016 in both countries (observed effect: 19% relative reduction in HIV incidence [95% CI 1–34]^10^); the model predicted a relative reduction in HIV incidence of 28% (95% CrI 14–39) in a population cohort-like population and 33% (22–44) when examining the effect on the whole community (figure 2).

Under scenario 1, the PopART intervention was consistently predicted to lead to at least 50% lower HIV incidence in the whole community on average than standard of care across all arms of the trial when continued over 2020–30 (arm A: 51% relative reduction in HIV incidence [95% CrI 34–62]; arm B: 51% [34–64]; post hoc arms A and B combined: 51% [40–60]; figure 2).

Before data unblinding, the model struggled to reproduce intervention effects in arm A for a number of reasons. First, because we calculated intervention effect using the paired counterfactual simulations, in which non-intervention model parameters were the same, and hence HIV incidence was identical until the beginning of the trial, the uncertainty bounds in the estimated effect of the trial were decreased compared with the real-world comparison with arm C (appendix pp 6, 29). Second, poor fit to sex-specific viral suppression data (only available after unblinding) meant two sex-specific parameters within the cascade were introduced to the model after unblinding. Third, the scarcity of cascade data in South Africa before data unblinding meant data from the end of the trial were the earliest piece of data used to determine engagement with the care cascade at trial baseline.

**Figure 3:**
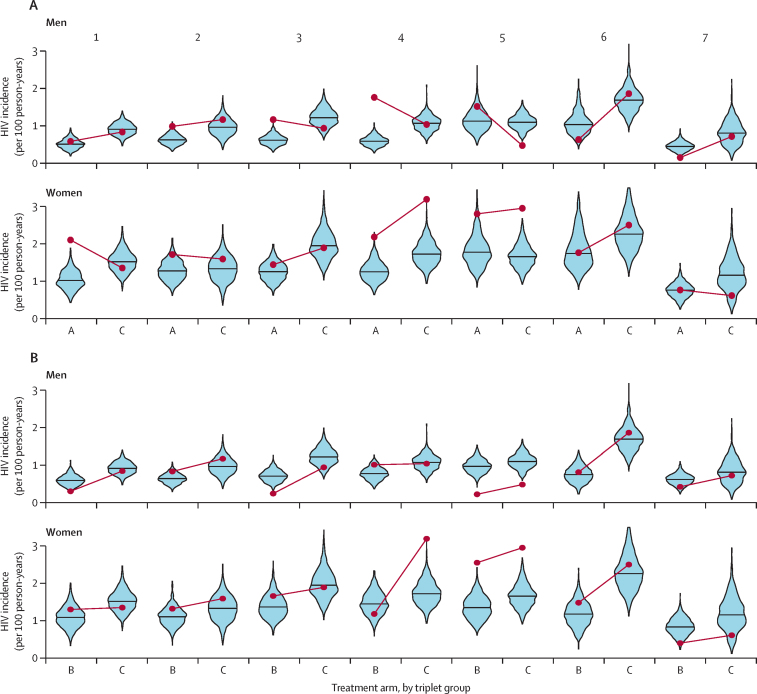
Predicted average HIV incidence rate using post-unblinding model versus observed HIV incidence over population cohorts 12–36 (A) Arm A *vs* arm C communities. (B) Arm B *vs* arm C communities. Projections are stratified by sex across seven triplets, across 1000 parameter sets. Horizontal lines within violin plots show medians of model projections.

Projections of HIV incidence disaggregated by community and sex in the post-unblinding model show large variation across communities (figure 3; appendix pp 8–9). Overall, the post-unblinding model performed well at predicting HIV incidence stratified by sex in arms B and C, and consistently underestimated HIV incidence in arm A compared with observed trial data (figure 3; appendix pp 9–11).

Overall, the model provided a reasonable fit to HIV prevalence and the HIV-care cascade data in all communities (appendix pp 32–33). The model consistently underestimated the proportion of individuals reporting as being on ART among those aware of HIV status, especially among women, perhaps indicating that observed self-reported ART status is biased.

No parameter was highly correlated with predicted HIV incidence over population cohort 12–36, although some parameters were weakly correlated with HIV incidence in each of the communities (eg, average annual hazard of HIV infection and the multiplier to account for potential over-reporting or under-reporting of number of sexual partners). Community-level projections of prevalence of detectable viraemia showed a strong correlation with predicted HIV incidence, consistent with observational studies (appendix pp 13–14).^22^

Several calibrated parameters were correlated, such as those governing partnership formation (multiplier on number of partners, and risk assortative mixing—ie, the propensity for individuals to form partnerships with others of the same sexual risk group) and the overall risk of HIV acquisition in a serodiscordant partnership (appendix pp 32–33).

We projected several secondary endpoints with the post-unblinding model. Projected mean HIV-related mortality decreased by 30–40% between 2014 and 2017 in arm A and B communities (appendix pp 14–16) compared with a range of 0–15% mean decrease in arm C communities. HIV-related mortality is predicted to decrease to 2030 under continuation of the PopART intervention, and under standard of care in arm C communities, with steep decreases associated with effects of the PopART intervention in the intervention communities (appendix pp 14–16).

The median proportion of HIV incidence predicted to be attributable to the acute and early phase of HIV infection (which was modelled uniformly as 1–3 months after infection) ranged from 11% to 15% across all communities in 2020 (appendix pp 16–18). This proportion was projected to increase over time if a PopART intervention was continued (scenario 1), ranging from 11% to 22% in 2030 (appendix pp 16–18). This proportion decreased with increasing age due to two factors: engagement with the care cascade increases with age, and rate of partnership change decreases with age. This proportion was higher in men than in women, most acutely in the youngest 5-year age group, due to sexual mixing patterns, meaning women seem to usually form partnerships with men older than themselves. Older men have a higher chance of being HIV positive for a longer period of time than do other age and sex groups (appendix pp 16–18).

VMMC uptake was modelled as being the same across communities. Traditional male circumcision varied greatly across communities and drove differences in total proportion of men receiving VMMC in the model (appendix pp 19–20).

In three triplets (1, 2, and 5), HIV incidence as measured in population cohort 12–36 was unexpectedly found to be higher in arm A than in arm C.^10^ Using the post-unblinding model, the model predicted this dissonance in triplet 5, resulting from lower awareness of HIV status for both men and women across all age groups in triplet 5 arm A than in triplet 5 arm C at trial baseline (appendix pp 20–21, 32).

**Figure 4:**
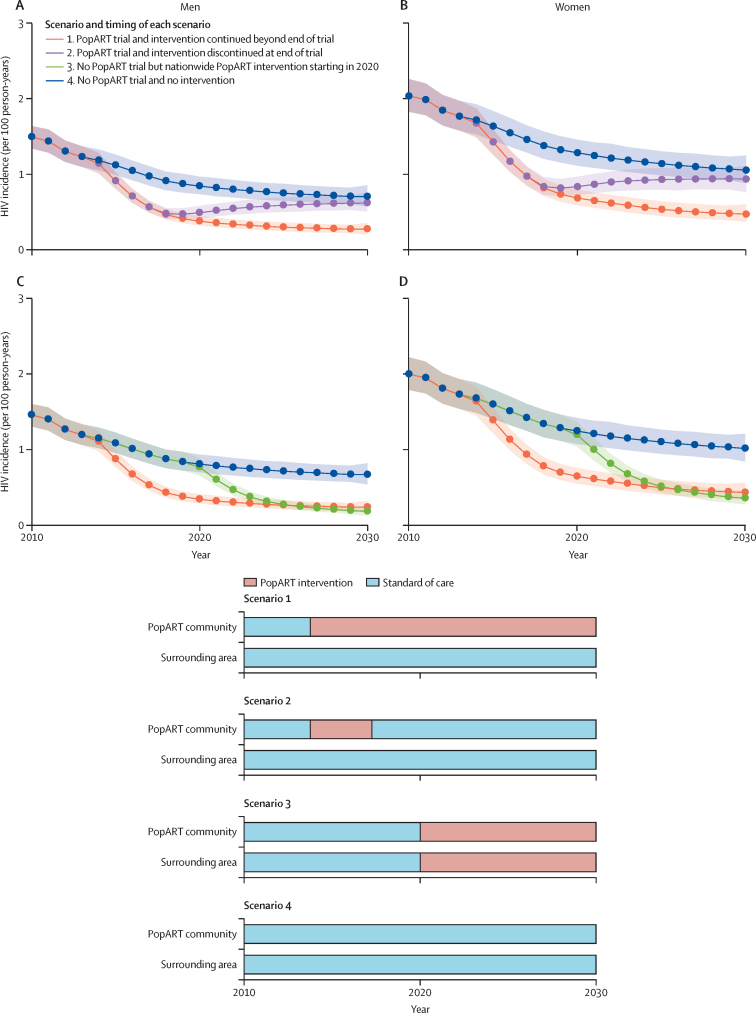
Projected mean HIV incidence across total population of arm A and B communities for the period 2010–30 For ease of comparison the top row (A, B) shows scenarios 1, 2, and 4, and the bottom row (C, D) shows scenarios 1, 3, and 4. Solid lines show the median of the distribution of the arithmetic mean of HIV incidence per 100 person-years across all intervention communities and shaded areas show 95% credible intervals of mean HIV incidence. Median and 95% credible intervals are across model output from 1000 parameter sets that have been randomly drawn from each community.

When modelling projections of the PopART intervention up to 2030, we predicted a declining trend in HIV incidence in both males and females under all modelled scenarios, including standard of care, and projections showed greater reductions in annual HIV incidence in 2030 when the PopART intervention was continued for a longer period of time (figure 4). None of the projected scenarios reached the suggested milestone of so-called epidemic control of HIV incidence below 0·1 per 100 person-years by 2030.^23^

Under a scenario of a continued PopART intervention in the intervention communities (scenario 1), HIV incidence was projected to be 58% lower in 2030 than in a counterfactual scenario (scenario 4; from 0·86 [95% CrI 0·75–1·00] to 0·36 [0·30–0·44] cases per 100 person-years; percentage reduction in incidence of 55% in women and 61% in men; figure 4; appendix p 22). HIV incidence was projected to be reduced by 30% in 2030 compared with 2020 (from 0·52 [95% CrI 0·45–0·61] to 0·36 [0·30–0·44] cases per 100 person-years; 31% in women and 28% in men; figure 4; appendix pp 21–22). Discontinuation of the PopART intervention after the end of the trial (scenario 2), which is the current situation in the trial communities, was projected to result in an overall reduction in HIV incidence of 11% by 2030 (from 0·86 [95% CrI 0·75–1·00] to 0·77 [0·66–0·89] cases per 100 person-years; 11% in women and 11% in men) compared with the counterfactual scenario (scenario 4; figure 4; appendix p 22).

Introducing the PopART intervention widely to all areas in 2020 (scenario 3) is projected to lead to HIV incidence reduction of 66% in 2030 compared with the counterfactual scenario (0·86 [95% CrI 0·75–1·00] to 0·29 [0·24–0·35] per 100 person-years; percentage reduction in incidence of 64% in women and 69% in men) compared with a scenario of no community HIV-care providers roll-out in 2030. By 2025, this scenario is projected to surpass reductions in the scenario in which the PopART intervention was only provided to an individual community from 2014 onwards (scenario 1; figure 4; appendix pp 21–22). Under counterfactual simulations (scenario 4), HIV incidence is projected to be reduced by 18% by 2030 compared with 2020 (from 1·05 [95% CrI 0·92–1·19] to 0·86 [0·75–1·00] per 100 person-years; 18% in women and 17% in men; figure 4; appendix pp 21–22).

By 2030, the 95-95-95 targets were projected to be met when taking mean projections across all intervention communities under the scenario of a continued PopART intervention after the end of the trial (scenario 1; appendix p 22). But not all communities are projected to reach 95-95-95 by 2030 and no scenario investigated met the 95-95-95 targets in every community by 2030.

The reproduction number (R) of the epidemic in each community, summarising the average number of onwards transmissions for each incident case, was projected to be less than 1 over the whole simulated period under a scenario of a continued community HIV-care providers roll-out after the end of the trial (scenario 1), or a scenario of roll-out of community HIV-care providers to a wider area in 2020 (scenario 3; appendix pp 18–19). In all counterfactual simulations, and under a scenario of discontinuation of community HIV-care providers, R is projected to settle very close to 1 by 2030.

## Discussion

As part of the HPTN 071 (PopART) trial, a cluster-randomised trial of HIV prevention, we present modelling projections of intervention effect on HIV incidence in 21 high prevalence communities in South Africa and Zambia. The model had good predictive ability in arm B and poor predictive ability in arm A; the model could explain higher incidence in arm A than in arm C in one of three of the triplets where this effect occurred, resulting from consistently worse engagement across all age and sex groups in the care cascade in arm A at trial baseline (ie, mismatching). The PopART intervention is projected to reduce HIV incidence by approximately 51% (95% CrI 40–60) between 2020 and 2030 compared with standard of care.

Before data unblinding of the primary endpoint of the trial, the model prediction overlapped with the observed intervention effect in arm B but not in arm A. After data unblinding, the model prediction overlapped with the observed intervention effect in both arms over population cohort 12–36 (although the effect of the observed intervention effect in arm A was at the lower extreme of what the model simulated). The model provided reasonable fit to HIV incidence in arm B when disaggregated by community and sex. Projected to the whole community, intervention effect was expected to be 5–7% percentage points higher than in the population cohort population (age 18–44 years) because older age groups had higher ART coverage and viral suppression, and observed effect was higher in men (the population cohort comprised approximately 70% women).

We took measures to improve the credibility and efficiency of our analysis and protect against cognitive biases.^24^ For instance, an analysis plan for this modelling study was publicly pre-registered,^17^ the modelling team were masked to the data for the primary endpoint of the trial, interim analyses were presented to an independent DSMB throughout the trial, and trial projections were lodged in a data repository before data unblinding.

Here, we present projections of four future scenarios of UTT to 2030, which suggest that UTT can provide substantial reductions in HIV incidence. Continuing the PopART intervention was projected to reduce HIV incidence by 58% compared with standard of care by 2030 and is projected to reach the 95-95-95 targets by 2030. The model predicted decreases in HIV-related mortality between 30% and 40% over the 3-year trial period, in line with those observed in other UTT trials.^25^

Under a scenario of continuation of the PopART intervention, median projected HIV incidence was not projected to reach the milestone of so-called epidemic control (<0·1 per 100 person-years by 2030),^23^ consistent with findings of other modelling studies.^26^

The model projected an increasing contribution of transmission from the acute phase of HIV infection between 2020 and 2030, which increased with decreasing age and was higher in young men than in young women. However, most transmissions at all timepoints were projected to be from long-standing (ie, not acute) infections. These results suggest a need for frequent testing, to target transmission from acute infections, while adherence counselling, differentiated service delivery, and measures to reduce drop out also remain important to target transmissions from people with long-standing infections. This finding underscores the importance both of combination prevention, and of the universality of treatment as prevention across age and sex groups.

Correlations between calibrated parameters highlight two explanations for model dynamics: either an epidemic that involves individuals having riskier sexual practices per sexual act (eg, less condom use), or by individuals having a wider variety of sexual partners (with regards to sexual risk-taking behaviour). Additional data on sexual mixing and sexual behaviour, or model selection across different patterns of sexual partnership formation and dissolution, might resolve such uncertainties and improve model identifiability.

Modelling might help to explain the dissonant triplets observed in the trial, in which incidence was higher in arm A than in arm C.^10^ The model reproduced higher HIV incidence in arm A than in arm C in triplet 5, driven by differing estimates of the effect of standard of care before trial start, and illustrated in the observed data when compared at the community level. Modelling highlights that this dissonant arm A versus arm C effect in triplet 5 could be explained by poor matching on engagement in the HIV care cascade at trial baseline.

Overall, the model generally underestimated HIV incidence, and fitting the model directly to HIV incidence observed in the trial using the Approximate Bayesian Computation inference framework might improve estimates of drivers of differences between communities. The model did not explicitly include pre-exposure prophylaxis, drug resistance, different ART drug regimens, mother-to-child transmission, or migration (although partnerships between individuals in the PopART communities and the surrounding area were incorporated). Availability of data, and trade-offs between expected epidemiological importance, and computational complexity might restrict what processes are included in a model; however, there might be important processes that the model is not capturing and uncertainty regarding the structure of the model has not been taken into account in this analysis.

The proportion of individuals on ART among those aware of their HIV status is consistently underestimated in the model, particularly among women. The data used for fitting ART coverage is self-reported, which might be overestimated because of social desirability biases, especially in younger age groups,^27^, ^28^ although the Population-based HIV Impact Assessment surveys (known as PHIA surveys) suggest agreement between self-reported ART use and antiretroviral biomarkers.^29^ Given that the inference framework we used is triangulating between a range of data sources simultaneously, the model structure does not explicitly account for self-reporting bias of ART use, and has highlighted that the proportion of individuals on ART is inconsistent with other data sources (conditional on the structure of the model); therefore, model projections might give a more realistic estimate of ART coverage, implicitly accounting for self-reporting bias.

Projections in high HIV prevalence communities with a validated individual-based model estimate that a UTT intervention delivered by community HIV-care providers can reduce HIV incidence by over 50% by 2030 compared with standard of care. Projections to 2030 also predict substantial reductions in HIV-related mortality. We predict increasing importance of transmissions from the acute phase of HIV infection. Effects on HIV incidence are projected to be greater if the PopART intervention is introduced to a wider area, to minimise the occurrence of partnerships between individuals who are receiving the PopART intervention with those who are not. To date, UTT trials are likely to have underestimated the effect of interventions on HIV incidence as a result of movement into and across trial areas. Ultimately, this work provides evidence that combination prevention including UTT has the potential to have a major role in realising the UNAIDS 2030 targets.

For more on **UN mortality and fertility databases** see https://population.un.org/wpp/Download/Standard/Population/

## Data sharing

The HPTN 071 (PopART) data sharing policy is provided in the appendix (pp 42–47).

## Declaration of interests

AC reports funding from the National Institute for Health and Care Research (NIHR), Sergei Brin Foundation, and US Agency for International Development, and from Pfizer for lecturing. CF reports funding from the US National Institutes of Health (NIH), the National Institute of Allergy and Infectious Diseases (NIAID), the US President's Emergency Plan for AIDS Relief (PEPFAR), International Initiative for Impact Evaluation (3ie), the Bill & Melinda Gates Foundation, the National Institute on Drug Abuse (NIDA), and the National Institute of Mental Health (NIMH). DM reports funding from NIH, 3ie, PEPFAR, and the Bill & Melinda Gates Foundation. DJD reports funding from NIH and participation on a DSMB for COVID-19 studies. EP-M reports funding from NIH. HA reports funding from NIH, 3ie, and PEPFAR. MP reports funding from the Bill & Melinda Gates Foundation. SFl reports funding from NIH, 3ie, PEPFAR, and the Bill & Melinda Gates Foundation. TS reports funding from NIAID/NIH. WJMP reports funding from Li Ka Shing Foundation and is a consultant with WHO. All other authors declare no competing interests.
