# Supplementary material for: Projected outcomes of universal testing and treatment in a generalised HIV epidemic in Zambia and South Africa (the HPTN 071 [PopART] trial): a modelling study
Source: Lancet HIV. 2022 Nov 1;9(11):e771–80. doi: 10.1016/S2352-3018(22)00259-4 (PMC9646978; doi:10.1016/S2352-3018(22)00259-4)
Supplement: Supplementary appendix [file mmc1.pdf]

THE LANCET
HIV

Supplementary appendix

This appendix formed part of the original submission and has been peer reviewed. We post it as supplied by the authors.

Supplement to: Probert WJM, Sauter R, Pickles M, et al. Projected outcomes of universal testing and treatment in a generalised HIV epidemic in Zambia and South Africa (the HPTN 071 [PopART] trial): a modelling study. *Lancet HIV* 2022; **9:** e771–80.

Supplementary Materials

This document outlines the Supplementary Materials for "Prevention outcomes from universal testing and treatment in a generalised HIV epidemic in Zambia and South Africa (the HPTN 071 (PopART) trial): a modelling study".

TABLE OF CONTENTS

PRIMARY ENDPOINT (INTERVENTION IMPACT) AND HIV INCIDENCE 3
- HIV INCIDENCE 3
- COUNTERFACTUAL SIMULATIONS 3
- INTERVENTION IMPACT 4
- INTERVENTION IMPACT IN WHOLE POPULATION VERSUS POPULATION COHORT 7
- HIV INCIDENCE OVER PC12-36, OBSERVED VERSUS PREDICTED 7

SECONDARY ENDPOINTS AND OTHER INDICATORS 13
- PREVALENCE OF DETECTABLE VIRAEMIA (PDV) 13
- HIV INCIDENCE VERSUS PREVALENCE OF DETECTABLE VIRAEMIA 13
- HIV-RELATED MORTALITY 14
- NOTES ON CD4 PROGRESSION 16
- TRANSMISSION DURING EARLY AND ACUTE HIV INFECTION (AEHI) 16
- REPRODUCTION NUMBER, R 18
- VOLUNTARY MEDICAL MALE CIRCUMCISION (VMMC) 19
- LOSS-TO-FOLLOW-UP/DROPOUT AND TREATMENT FAILURE 20

INSIGHT INTO THE DISSONANT TRIPLETS 20

OUTCOMES IN MODELLED SCENARIOS, 2010-2030 21

INFERENCE FRAMEWORK AND PARAMETERISATION 23
- CHIPS TESTING COVERAGE 24
- STATISTICAL MODEL OF VIRAL SUPPRESSION 26
- INFERENCE FRAMEWORK 27
- UNBLINDING PROCESS 28
- MODEL FIT 32
- MARGINAL POSTERIOR DISTRIBUTIONS OF FITTED PARAMETERS 33
- PARAMETER CORRELATIONS 33

CODE AND ANALYSES 35
- CODE ORGANISATION AND DEPLOYMENT PIPELINES 35

GLOSSARY 36
- NOTE ON TERMINOLOGY 38

REFERENCES 38

HPTN 071 STUDY TEAM 41

HPTN 071 (POPART) DATA SHARING POLICY 42
- BACKGROUND 42
- TYPES OF HPTN 071 (POPART) DATA ADDRESSED BY THIS POLICY 42
- PRINCIPLES FORMING THE BASIS OF THE HPTN 071 (POPART) DATA SHARING POLICY 43
- KEY ASPECTS OF THE HPTN 071 (POPART) DATA SHARING POLICY 43

STORAGE FOR DATA SHARING....45
STANDARDS FOR DATA SHARING....45
CONDITIONS OF ACCESS FOR NON-PROTOCOL TEAM MEMBERS....46

Primary endpoint (intervention impact) and HIV incidence

HIV incidence

Average HIV incidence rate (*HIV.I.*) between time point *t0* and *t1* is defined as:

$$HIV.I^{d}{}_{c}(t_0, t_1) = \frac{I_c{}^{d}(t_0,t_1)}{N_c{}^{d}\ \ (t_0,t_1)},$$

where $I^{d}{}_{c}(t_0, t_1)$ is the number of new, incident HIV infections between time *t0* and *t1*, and $N^{d}{}_{c}(t_0, t_1)$ is the number of observed at-risk person-years between *t0* and *t1* in the simulated data. Subscript *c* refers to the study arm type for which HIV incidence is measured $c \in \{A, B, C, CF\}$ for communities in study arm A, B, C, or in the simulated counterfactual (CF; also a simulation of standard-of-care), respectively. The superscript *d* refers to the combination of data and model used to make the projections, $d \in \{pre, post\}$ for pre- or post-unblinding simulations, respectively. When *t1 - t0* is a short period of time, such as a single timestep (typically 1/48th of a year but may be 1/12th of a year in files used to calculate intervention impact), then it is assumed HIV incident cases occurring over the period *[t0, t1)*, contribute (*t1-t0*)/2 person years of at-risk observation time. Units for HIV incidence are expressed "per 100 person-years" throughout this analysis.

Where HIV incidence has been reported as an aggregation across multiple communities, such as in figure 4, the arithmetic mean across communities is taken (for 1000 bootstrap samples of output from the 1000 'accepted' parameter sets) and credible intervals (2.5% and 97.5% quantiles) are presented of the resultant 1000 mean incidence estimates. The model provides HIV incidence estimates conditional on a community-specific parameter set, $p_{y,i}$, for community *y* (since the model is calibrated separately for each community; $i \in 1, \ldots, 1000$) which are averaged across the communities of interest (typically all intervention communities, but the context may vary). That is, $\frac{1}{|C|}\sum_{c \in C} HIV.I^{d}{}_{c,p_{y,i}}(t_0, t_1)$, where *C* is the set of all communities in intervention arm of type *c. Where* $HIV.I^{d}{}_{c,p_{y,i}}(t_0, t_1)$ is the projected average HIV incidence rate between times t_0 and t_1 from parameter set $p_{y,i}$ in community *c* using data and model *d* (e.g. the pre-unblinding or post-unblinding model). Community number (*y*) and study arm type (*c*) use different subscripts here since the model can be run under different arms for different community numbers (although the set of community numbers will typically be dictated by study arm type given the trial design).

Counterfactual simulations

Counterfactual simulations were performed for some intervention communities. Counterfactuals in this analysis are simulations of the same intervention communities using the same parameter sets but without simulating the PopART trial or PopART intervention. Intervention communities were fitted to data where the intervention was taking place (i.e. data from the CHiPs). The model simulates both the PopART intervention and a standard-of-care concurrently in these communities (as would have taken place in reality), and the model therefore has to reconcile CHiPs data on interaction with the care cascade with how this may be apportioned between individuals whose interaction with the HIV care cascade was initiated via standard-of-care or via a CHiPs visit. The model largely reconciles this using data on the age- and sex-stratified testing coverage of CHiPs visits. In intervention communities the model fitting process still provides an estimate of standard-of-care in each of the intervention

communities because several parameters associated with standard-of-care are allowed to vary in the inference framework. It is therefore possible, in the intervention communities, to generate a simulation of standard-of-care using these counterfactual simulations by simulating intervention communities without the PopART intervention or PopART trial. This is mainly needed when there is no data to fit to arm C communities (because it is still necessary to generate a comparison within a triplet to estimate the impact of the intervention). Therefore, all calculations of intervention impact before data unblinding (in December 2018) were compared against counterfactual projections and all calculations of intervention impact after trial unblinding were compared against arm C projections. Scenario 4 in the modelled scenario section represent a simulation of standard-of-care so although model fitting to arm C communities never included simulation of the PopART intervention (because CHiPs data do not exist in these communities) sometimes arm C projections are presented as "counterfactual" projections from the intervention communities because they are all simulating the same representation of the standard-of-care, albeit estimated in different ways.

Intervention impact

As reported in the *HPTN 071 Individual-based Model Analysis Plan* (Probert, Sauter et al., 2018), intervention impact is reported as the incidence rate ratio (RR) (or relative HIV incidence) between intervention and control arms and as the relative reduction in HIV incidence (1 - RR).

The incidence rate ratio (RR), or relative HIV incidence, is computed as:

$$RR^{d}{}_{c,c'}(t_0,t_1) = \frac{HIV.I_c{}^{d}(t_0,t_1)}{HIV.I_{c'}{}^{d}\ (t_0,t_1)}$$

where *c* is either a community in arm A or B, and *c'* is a community in arm C or a CF scenario. All comparisons are made between communities in the same triplet (that is, *c* and *c'* are in the same triplet and represent a matched pair of communities) according to the HPTN 071 (PopART) trial study protocol.

The relative reduction in HIV incidence, *r*, over period *[t0, t1)* for treatment arm *c* (compared to *c'*) is computed as:

$$r\ {}^{d}{}_{c,c'}(t_0,t_1) = 1 - \left(\prod_{c\in C} RR^{d}{}_{c,c'}(t_0,t_1)\right)^{1/|C|}$$

Where *C* is the set of all communities in intervention arm of type *c.* That is, the geometric mean of pairwise rate ratios. Where the combined effect of arms A & B are estimated together, *C* is the set of all A and B communities (|*C*| = 14).

Model output is generated for 1000 parameter sets, as output from the inference framework (below), so the aggregate measures above are calculated for bootstrap samples of size 1000 of model output, giving distributions of the above values. Where intervention arms are matched with an in-silico self-counterfactual (that is, arm A compared to a counterfactual of arm A with the same parameters but without simulation of the trial), the measures above were also calculated for counterfactuals that had the same parameter sets (the only difference being the simulation of the PopART intervention). Unless stated explicitly, calculations of relative incidence are not matched on the parameter set used.

Intervention impact is calculated for the following time frames: PC0-PC36, PC0-PC12, PC12-PC24, PC24-PC36, PC12-PC36, 2020-2030 (table S1). Intervention impact is also calculated for different populations: the whole simulated population, a 'PC-like' population following the age-range of the PC Cohort (18-44 years old),) and using a weighting following the gender-distribution in the PC Cohort.

Table S2 shows the details of the cohort (age/sex characteristics), observation period, and comparison used for the four calculations of the intervention impact shown in figure 2 in the main text.

Counts in the simulated data (the whole simulated 'population') were first weighted according to the proportion of each gender observed in the PC. In Zambian communities this was 27.29% men and in the South African communities this was 30.69% men.

Period/Round	Start date	End date
PC0	15 January 2014	31 March 2015
PC12	01 July 2015	30 June 2016
PC24	06 August 2016	14 July 2017
PC36	08 September 2017	30 June 2018
2020-2030	1 January 2020	1 January 2030

Table S1: Timings of rounds of the Population Cohort as used in the individual-based model. Numbers in period/round name are months after trial start (e.g. PC12 is 12 months after trial start).

**Name**	**Age range**	**Proportion male**	**Period**	**Comparison**
PC12-36, paired counterfactual comparison	18-44	0.27	12-36 months	Counterfactual
PC12-36 arm C comparison	18-44	0.27	12-36 months	Arm C
Whole population, PC12-36, arm C comparison	13-80	0.497	12-36 months	Arm C
Whole population, 2020-2030, paired counterfactual comparison	13-80	0.497	2020-2030	Counterfactual

Table S2: Parameters of modelling projections of the primary endpoint of the HPTN 071 (PopART) trial as used in the projections of intervention impact in figure 2 (of the main text).

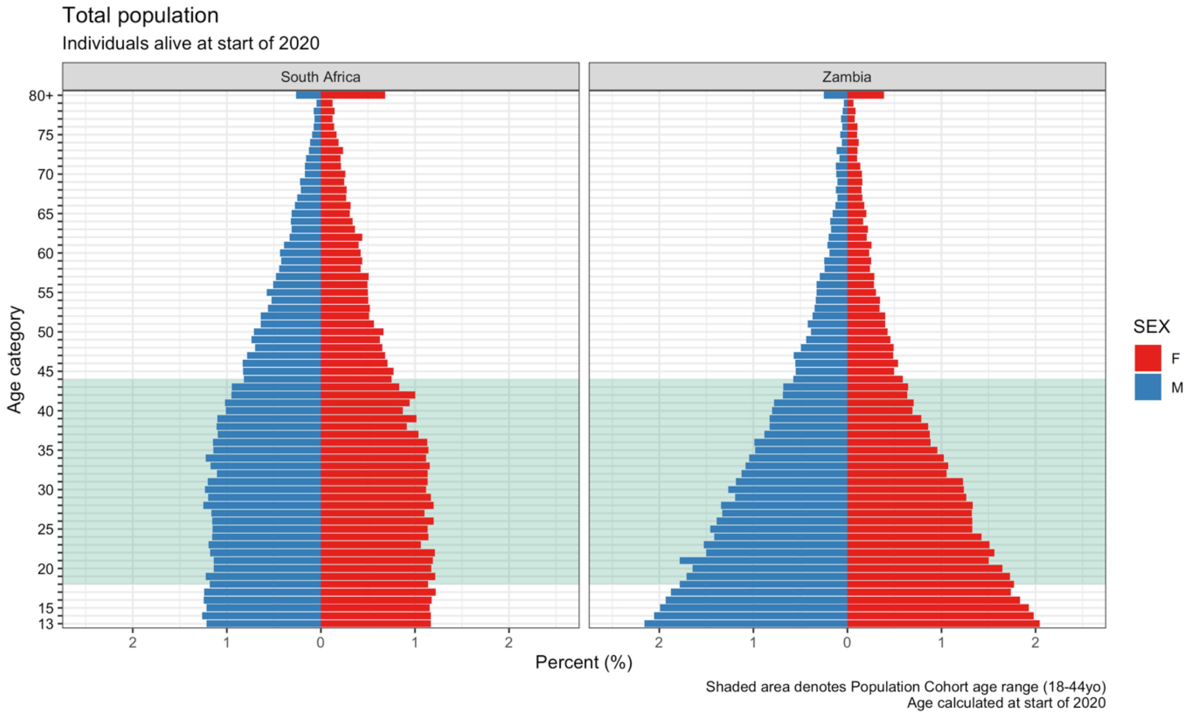

Figure S1: Age pyramid of a single simulated population in 2020. Green shaded area denotes ages in the Population Cohort (18-44yo).

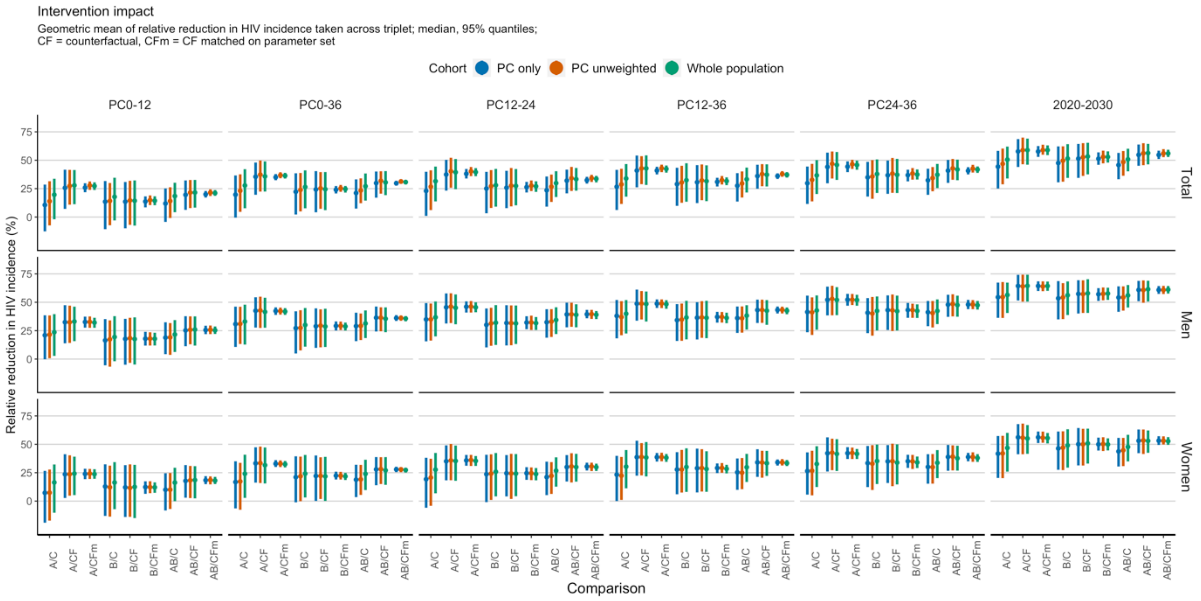

Figure S2: Relative reduction in HIV incidence (intervention impact) across all triplets, all time periods of interest (panel columns), three cohorts (PC gender-weighted, PC age-range but unweighted by gender, whole simulated population), and 9 possible comparisons (intervention vs counterfactual (pairing intervention and counterfactual randomly), intervention vs paired counterfactual, and intervention vs arm C; where intervention can be arm A, arm B, or arms A&B combined). Rows are total and then for men and women. Geometric mean of relative HIV incidence is taken across triplets. Results summarise median and 95% credible intervals from 1000 parameter sets as output from the inference framework. All results are from the model calibrated to trial data after trial unblinding. The predicted impacts over 2020-2030 in this figure relate to the modelled scenario 1: a scenario of the PopART trial and continued PopART intervention in the intervention communities.

Intervention impact in whole population versus Population Cohort

The intervention impact was consistently reported as being higher in the whole population compared to the Population Cohort. This can be explained by the differences in composition of age and sex in these two cohorts. The intervention had a larger impact in older age groups where uptake of the intervention was higher - older individuals reported higher ART coverage and higher levels of viral suppression, compared to younger individuals. The Population Cohort was approximately 70% women and the observed impact was also higher in men because their female partners had higher levels of ART coverage and viral load suppression. Comparing intervention and control arms over cohorts with an increasing upper age bound would therefore show an increasing impact. Such higher levels of engagement with care are reported in Floyd et al., (2018) and the greater impact in older participants was reported in Hayes et al., (2019): “adjusted rate ratio in participants ≥25 years of age, 0.58 (95% CI, 0.43 to 0.76) than in younger participants (adjusted rate ratio in participants 18 to 24 years of age, 0.92; 95% CI, 0.70 to 1.20).”

HIV incidence over PC12-36, observed versus predicted

Unless specifically mentioning pre-unblinding projections, all model output is using the post-unblinding model. Unless specifically mentioning counterfactual comparisons, all comparisons are with arm C projections.

For the period of 12-36 months after the start of the trial, 14 of 21 communities had an observed HIV incidence between 2.5% and 97.5% quantiles of model output (figure S4). Pearson’s correlation coefficient between mean predicted and observed HIV incidence over PC12-36 was lowest in arm A communities: 0.70 (overall), 0.63 (arm A), 0.81 (arm B), and 0.72 (arm C) (figure S6). Relative HIV incidence over PC12-36 is predicted to be within the 2.5% and 97.5% credible intervals comparing arms A and C communities in three of seven triplets.

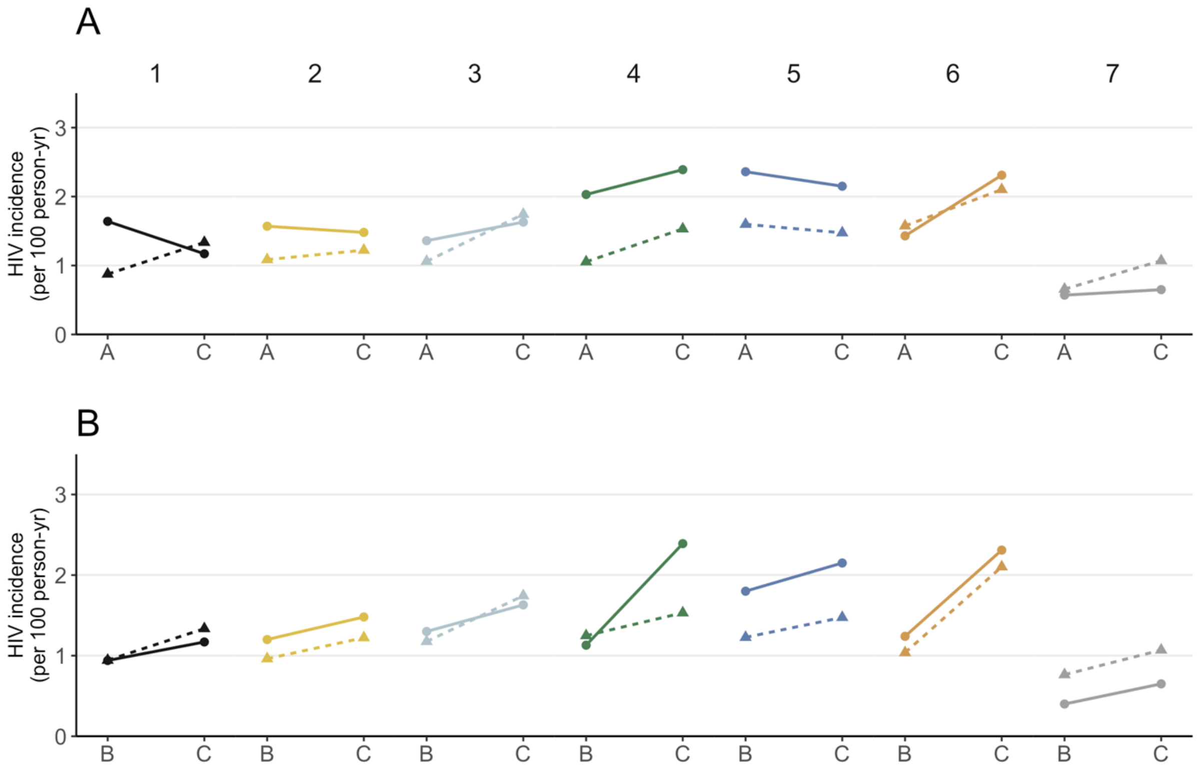

Figure S3: Predicted (dashed; mean) versus observed (solid) HIV incidence per 100 person-years (y-axis) over PC12-36 in A) arm A versus arm C communities and B) arm B vs arm C communities, stratified by arm comparison (panel rows), across 7 triplets (panel columns), and averaged across 1000 parameter sets. Observed data are from Hayes et al., (2019) and figure 3 shows this same data disaggregated by sex/gender. Figure S4 provides densities of predicted HIV incidence as violin plots.

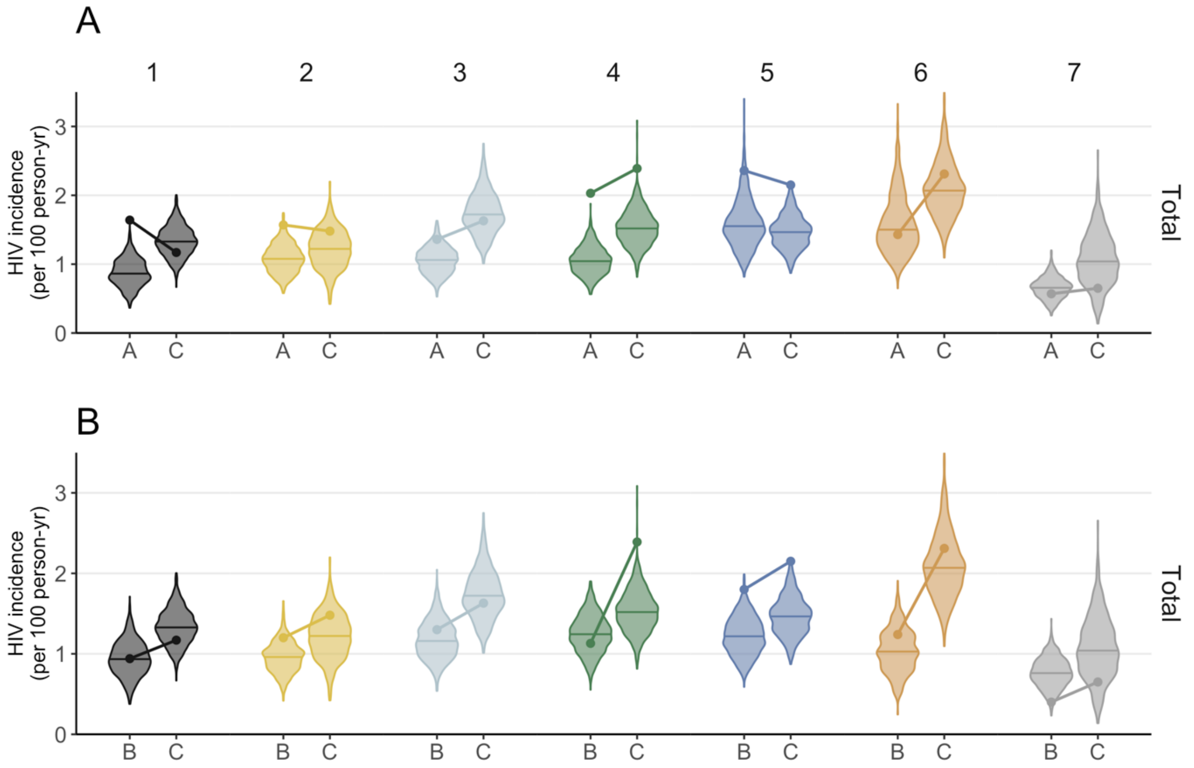

Figure S4: Predicted (violin plots) versus observed (solid) HIV incidence per 100 person-years (y-axis) over PC12-36 in A) arm A versus arm C communities and B) arm B vs arm C communities, stratified by arm comparison (panel rows), across 7 triplets (panel columns), and averaged across 1000 parameter sets for a PC-like population. Median shown as horizontal line of violin plots. Observed data are from Hayes et al., (2019) and figure 3 shows this same data disaggregated by sex/gender. Violin plots are mirrored kernel density estimates of the distribution of values limited to the range of the data. Triplets are coloured as in results of Hayes et al., 2019.

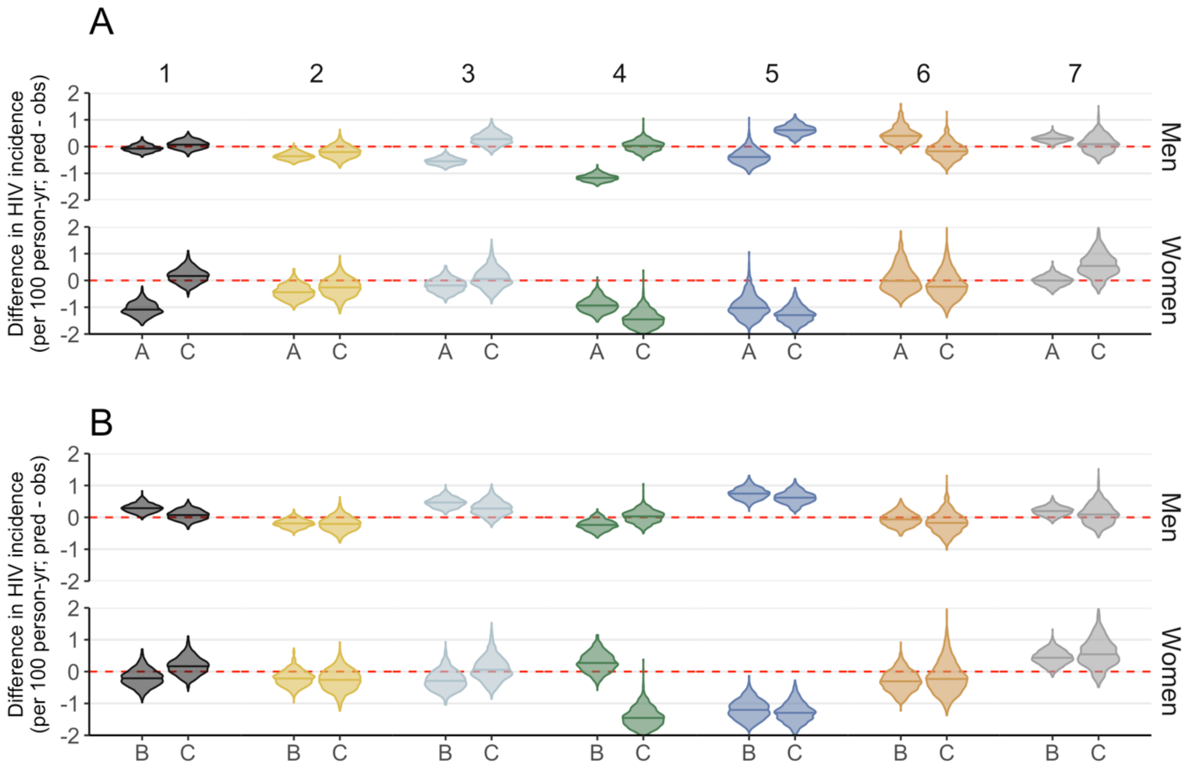

Figure S5: Absolute difference between predicted and observed HIV incidence over PC12-36 in a PC-like cohort, stratified by sex, across all triplets. Red dashed line shows a difference of zero. Medians are shown as horizontal lines in the violin plots. Violin plots are mirrored kernel density estimates of the distribution of values limited to the range of the data. Figure S5 shows the same data as figure 3 (main text) but as a difference between observed and predicted.

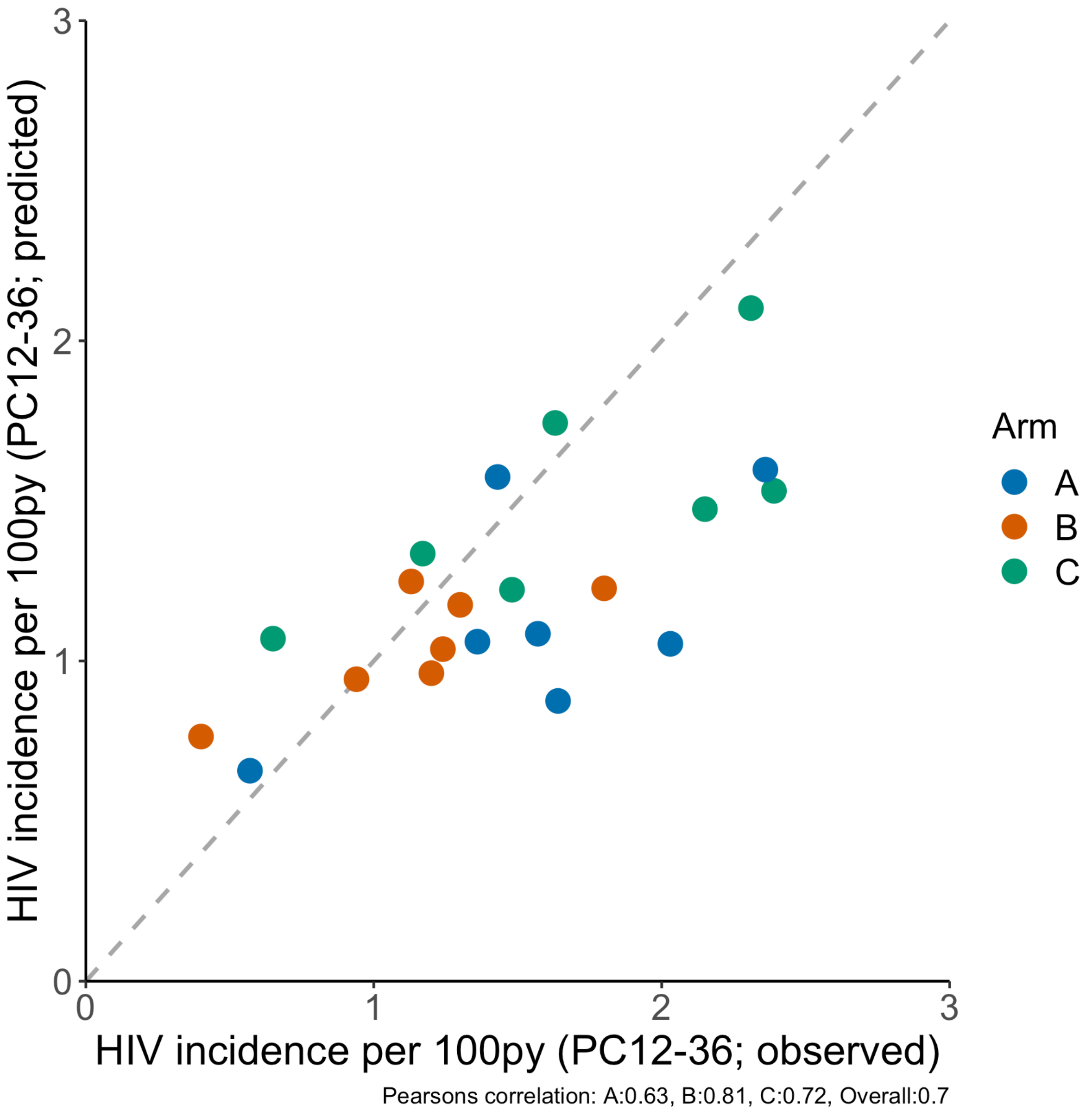

Figure S6: Observed versus predicted HIV incidence over PC12-36. Predicted incidence is the mean from 1000 simulations from calibrated output. Predictions are from the post-unblinding model calibration.

HIV incidence by age, 2010-2030

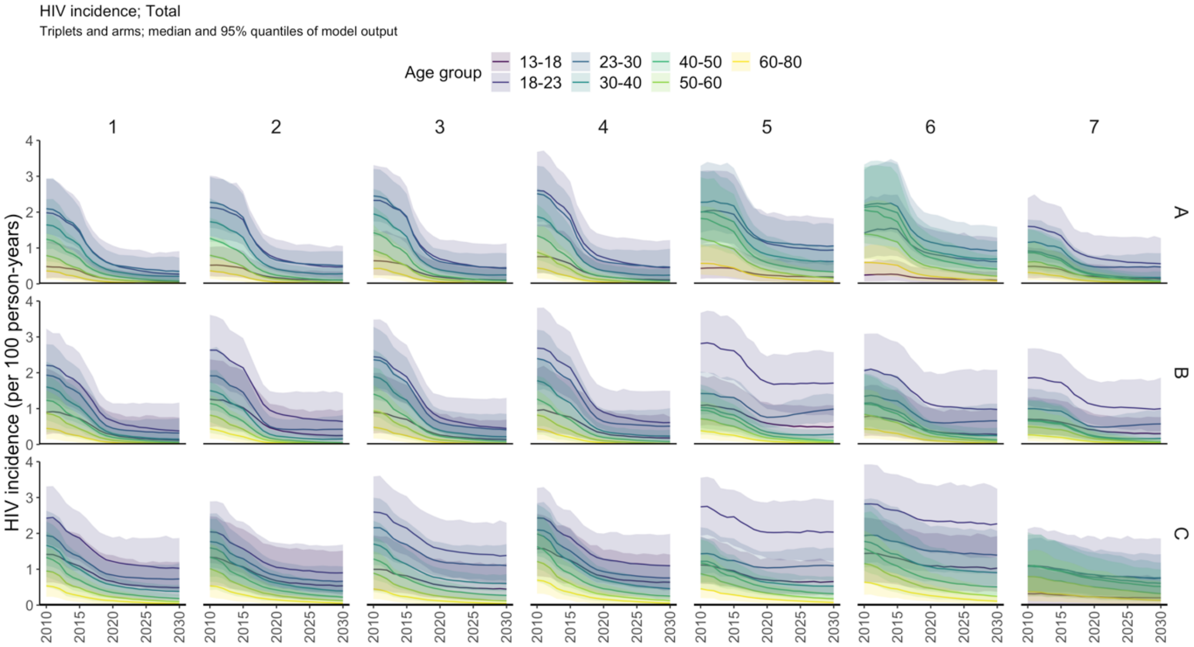

Figure S7: Projected total HIV incidence in each community by age (colours), triplet (columns), and arm (rows) from 2010 to 2030 under a scenario of continued PopART intervention after the end of the trial in intervention communities (scenario 1) and standard-of-care in arm C communities.

Secondary endpoints and other indicators

Prevalence of detectable viraemia (PDV)

The prevalence of detectable viraemia was projected to decline to 2030 and predicts higher PDV in women than men (figures S8-S9).

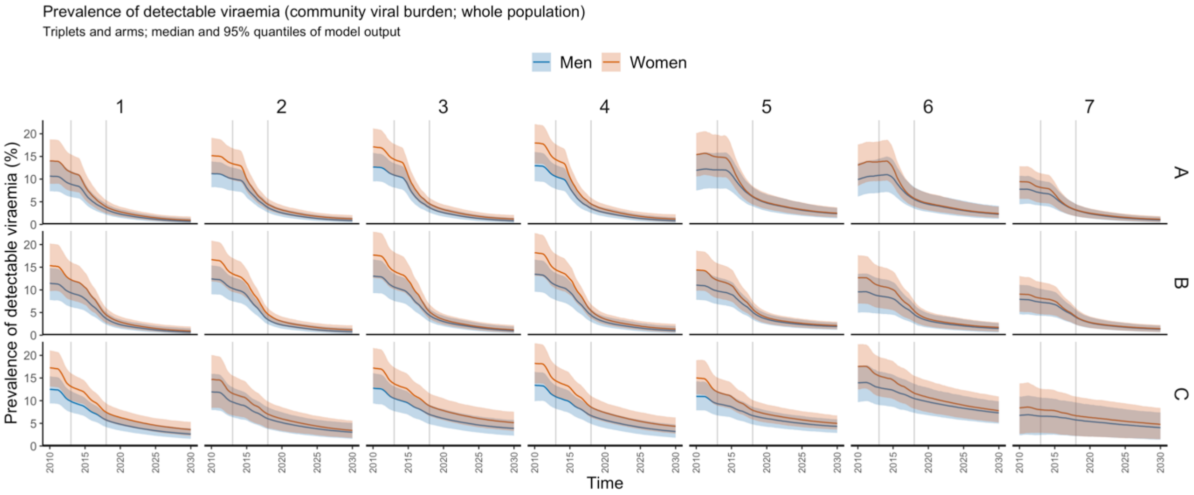

Fig S8: Predicted time-series of prevalence of detectable viraemia over 2010-2030 for men (blue) and women (red) in all triplets (columns) and arms (rows) of the trial. Shaded areas summarise 95% credible intervals from of model output from 1000 calibrated parameter sets. Vertical grey lines show approximate start and end of the trial. Simulations performed with post-unblinding model.

HIV incidence versus prevalence of detectable viraemia

Prevalence of detectable viraemia (PDV; sometimes called community viral burden) is calculated as the proportion of the population (including HIV negative individuals) that is viraemic (Herbeck and Tanser, 2016; Solomon et al., 2016). PDV is among three measures that have been found to be correlated with HIV incidence (Solomon, 2016): 1) “aware viral load”, the mean viral load from individuals aware of their diagnosis, whether in care or not); 2) “population viral load”, the mean viral load across all HIV-positive individuals, whether in care or aware of their HIV status or not); 3) and “prevalence of viraemia”, the prevalence of HIV-infected individuals with HIV viral loads greater than 150 copies per mL. We use the third metric definition, which we refer to as “prevalence of detectable viraemia”, but called “community viral load” in other studies, where simulated individuals are classified as virally unsuppressed according to the model definition.

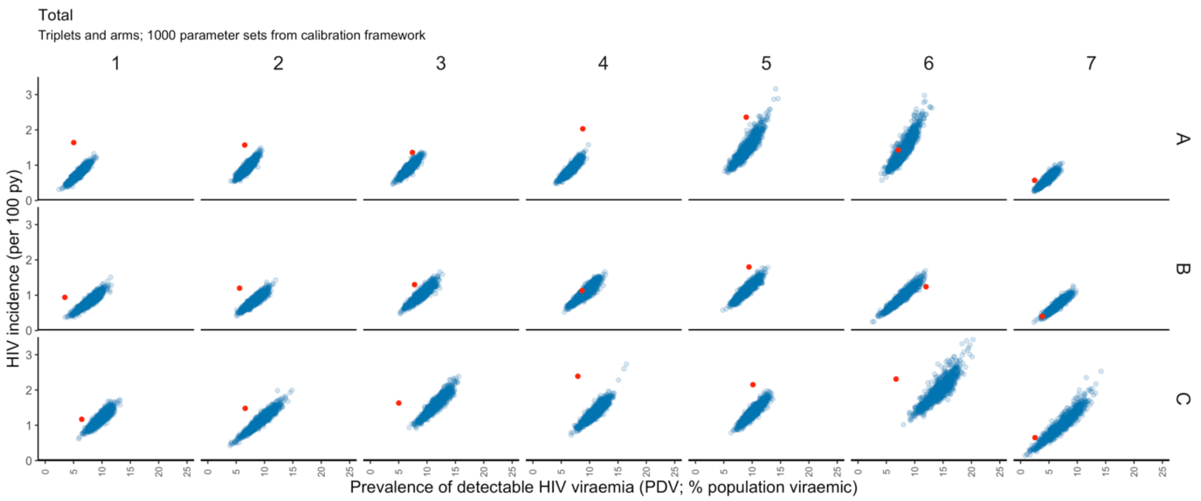

Fig S9: Scatterplot of prevalence of detectable viraemia in the community (PDV) at PC24 against HIV incidence over PC12-36 for a population of the PC age range across all arms (rows) and triplets (columns). Red dots show observed data.

HIV-related mortality

Figure S10 shows total HIV-related mortality through time. This indicator is similar to indicator BI.5 from the WHO Consolidated HIV Strategic Guidelines (WHO, 2020), with a numerator of estimated total number of people who have died from AIDS-related causes during the calendar year and denominator of total population regardless of HIV status. Deaths are also shown as percentage of total deaths, and absolute number of AIDS-related deaths in the simulated population (below). Projections are in line with previous studies that estimated 28.5% of deaths, on average, attributable to HIV/AIDS in 2017 in South Africa (figure S11; Gona et al., 2020).

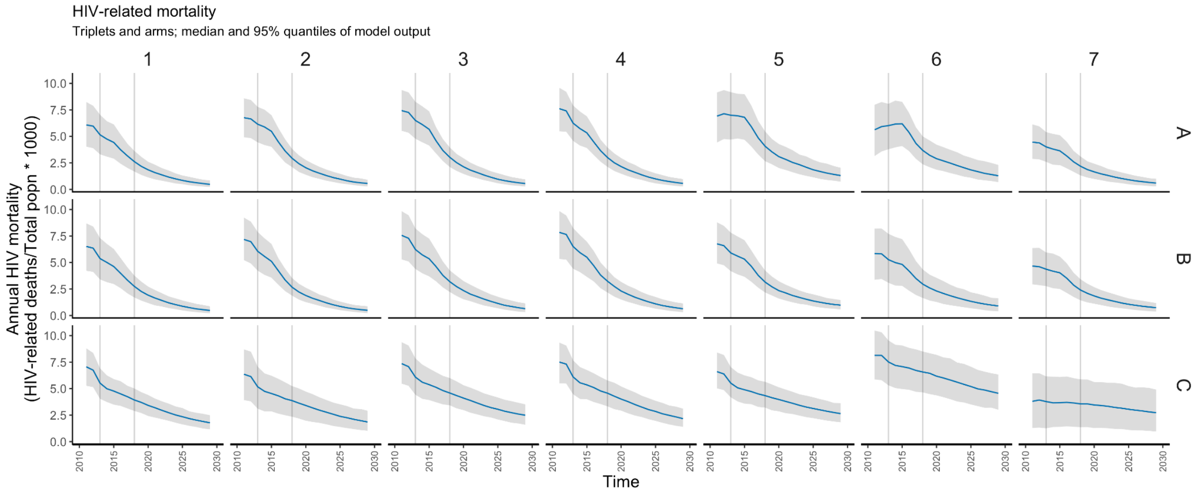

Fig S10: Predicted time-series of HIV-related mortality over 2010-2030 in all triplets and arms of the trial. Vertical grey lines show start and end of the trial. Beyond the end of trial, simulations assume continuation of a PopART intervention in the trial community, standard-of care, in the outside community. Mortality here is summarised in the modelled trial community only (‘inside’ patch; see model description).

Community	HIV-related mortality 2014	HIV-related mortality 2017	Percent change	Triplet	Arm
1	5.01	3.33	33.55	1	B
2	4.74	3.16	33.49	1	A
3	4.99	4.24	15.09	1	C
4	4.74	4.04	14.8	2	C
5	5.89	3.62	38.56	2	A
6	5.57	3.34	39.98	2	B
7	5.61	4.82	14.02	3	C
8	6.12	3.67	39.96	3	A
9	5.72	3.79	33.74	3	B
10	5.74	3.65	36.31	4	A
11	5.93	3.82	35.67	4	B
12	5.55	4.78	13.88	4	C
13	5.6	3.79	32.39	5	B
14	6.94	4.86	30.01	5	A
15	5.08	4.5	11.35	5	C
16	6.16	4.34	29.55	6	A
17	7.19	6.72	6.62	6	C
18	4.99	3.5	29.94	6	B
19	3.8	2.6	31.61	7	A
20	4.18	2.87	31.41	7	B
21	3.66	3.65	0.34	7	C

Table S3: Change in projected HIV-related mortality between 2014 and 2017.

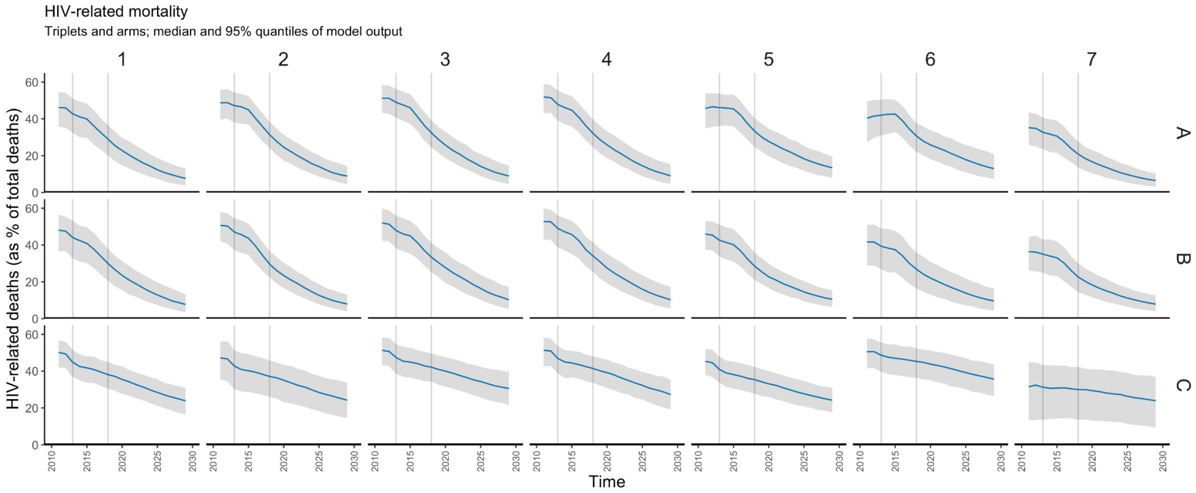

Fig S11: HIV-related deaths as a percentage of total deaths through time. Vertical grey lines show approximate start and end of the trial. Beyond the end of trial, simulations assume continuation of a PopART intervention in the trial community and standard-of care in the outside community (scenario 1).

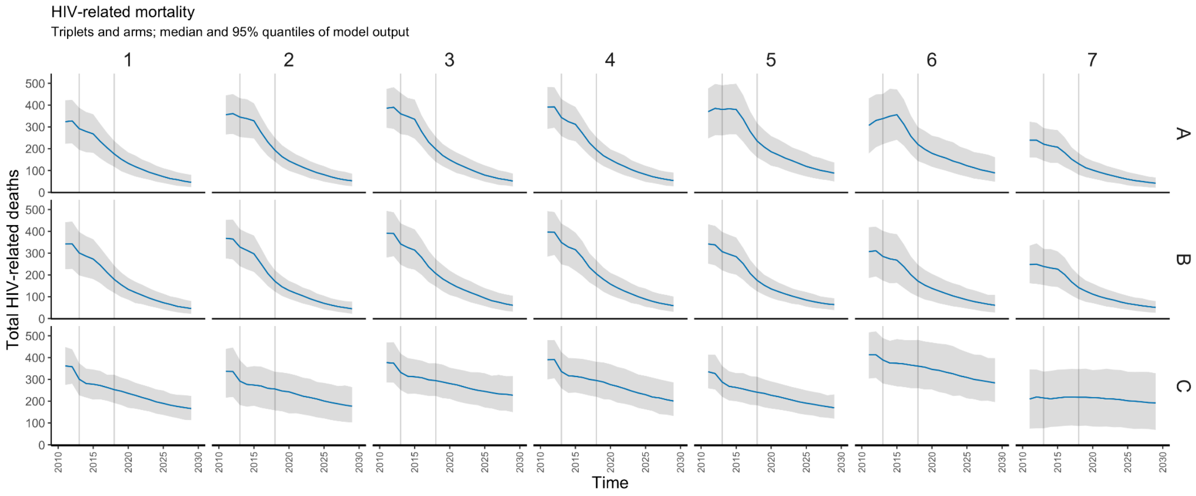

Fig S12: Total number of HIV-related deaths through time. Vertical grey lines show start and end of the trial. Beyond the end of trial, simulations assume continuation of a PopART intervention in the trial community and standard-of care in the outside community (scenario 1).

Notes on CD4 progression

The model uses CD4 progression derived from Cori et al., (2015) which is based upon the ATHENA cohort in the Netherlands. Findings from the ATHENA cohort was used in this modelling analysis because it represented a large dataset, covering treatment status, multiple CD4 count measurements, and set-point viral load (which were not available within South Africa and Zambia at the time the model was developed). The model does not account for host ethnicity or race in rate of CD4 decline but we note that in some studies this has been shown to be different (e.g. Easterbrook et al., 1996; Müller et al., 2009; Klein et al., 2014). Müller et al., (2009) and Klein et al., (2014) reported slower CD4 decline in patients of African descent, and Easterbrook et al., (1996) showed a slower CD4 decline in black compared to white homosexual and bisexual men in Baltimore, US. Mangal et al., (2017) used a large range of seroconverter data and did not find a difference in CD4 progression between African and European cohorts.

However, we do not report CD4 levels directly in our analyses. CD4 progression is used within the model for 1) initiating care before universal ART or outside arm A, 2) modelling disease progression, and 3) relative infectivity of individuals in different stages of CD4 decline (e.g. CD4 count of >500, 500-350, 350-200, <200 respectively). Regarding initiating care, the model is fitted directly to awareness of HIV status and ART coverage. In regard to relative infectivity, the model only assumes this is different when an individual's CD4 count is below 200 (Pickles et al., 2021). Regarding disease progression, we have reported that the simulated HIV-related mortality is similar to that in other studies (figure S11). We also note that studies have reported no statistical differences between risk of AIDS and death due to race/ethnicity (Silverberg et al., 2009 and references therein).

Transmission during early and acute HIV infection (AEHI)

Proportion of incidence attributable to acute phase of HIV infection (AEHI) is presented in the following three figures (disaggregated by age in the second and third). These figures highlight several important points. Firstly, this process is highly age dependent since the drivers are

also highly age dependent: interaction with care cascade, frequency of partner change, and coital frequency. Secondly, young men have a higher proportion of incidence attributable to the acute phase of HIV infection as men typically have heterosexual partners younger than themselves, there is higher coital frequency in younger ages, and interaction with care cascade is lower in young people. These results underscore the importance of frequent testing in younger age groups and adherence counselling in older age groups. Future projections highlight increases in the proportion of incidence from the acute phase in younger age groups. Demographic structure is likely to have a large impact but these two countries were similar in this respect in the model (figure S1).

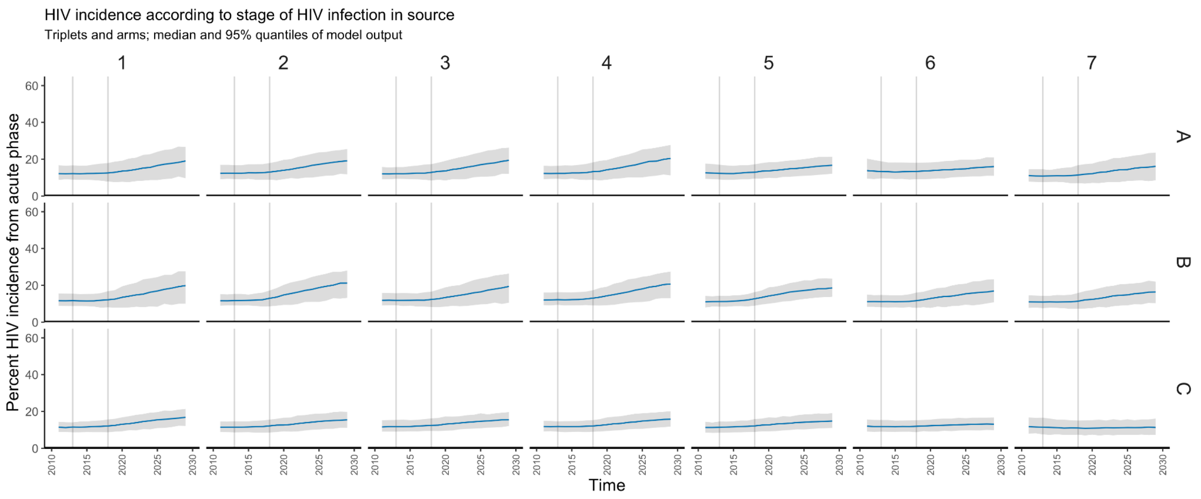

Fig S13: Predicted proportion of HIV incidence where the source individual was in the early and acute phase of HIV infection. Vertical grey lines show start and end of the trial. Beyond the end of trial, simulations assume continuation of a PopART intervention in the trial community and standard-of care in the outside community (scenario 1).

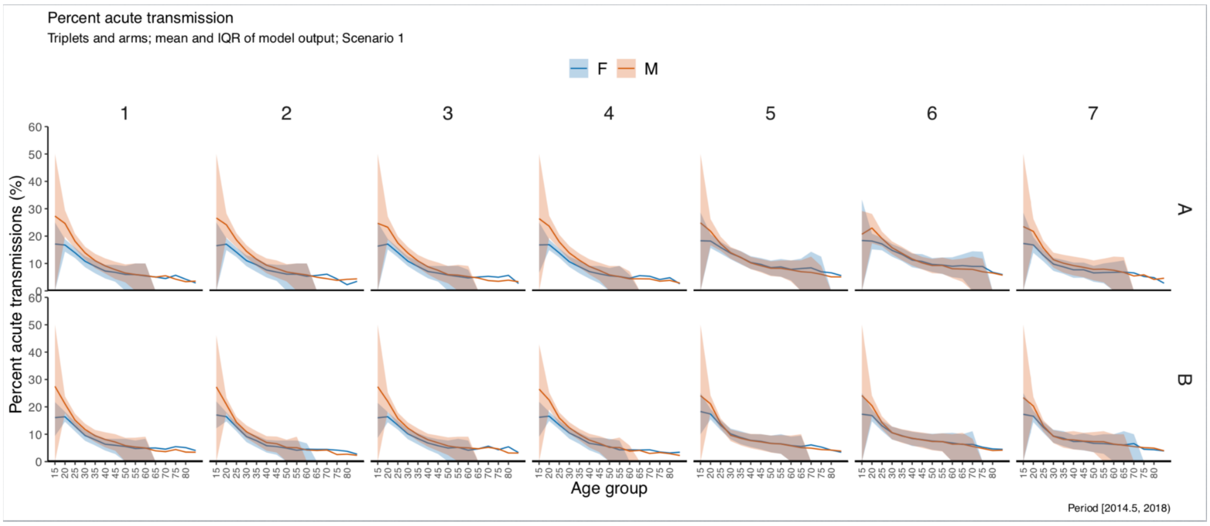

Fig S14: Predicted proportion of HIV incidence over the period [2014.5, 2018) where the source individual was in the early and acute phase of HIV infection, stratified by age and sex. Shaded areas show the interquartile range of model output. Shaded areas are not shown for some age groups because of small sample size (i.e. very few HIV incident infections in older age groups). Beyond the end of trial, simulations assume continuation of a PopART intervention in the trial community and standard-of care in the outside community (scenario 1).

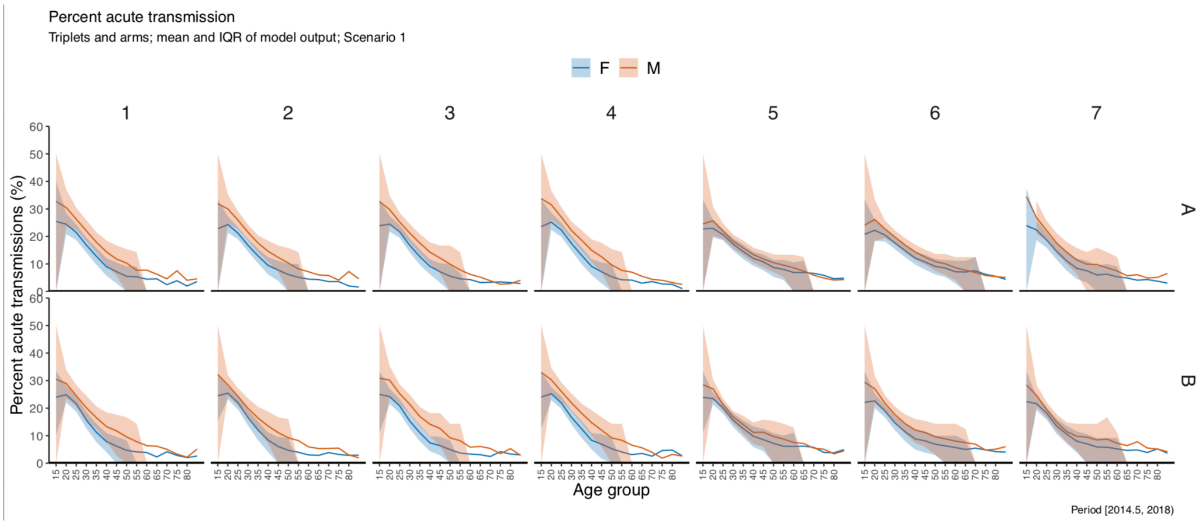

Fig S15: Predicted proportion of HIV incidence over the period [2025, 2030) where the source individual was in the early and acute phase of HIV infection, stratified by age and sex. Shaded areas show the interquartile range of model output. Shaded areas are not shown for some age groups because of small sample size (i.e. very few HIV incident infections in older age groups). Beyond the end of trial, simulations assume continuation of a PopART intervention in the trial community and standard-of care in the outside community (scenario 1).

Reproduction number, R

The reproduction number is calculated using the complete transmission tree. For each simulated individual living with HIV, the number of all future partners for which they are the source of HIV is recorded. The reproduction number shown is the average number of onwards transmissions for each source individual (shown at the time of infection of the source individual). Note that this method of calculating R is impacted by right censoring (i.e. the end time of the simulation as there are no simulated transmission events recorded beyond that time) and will show the impact of interventions slightly before they occur (if plotted against time of infection of source).

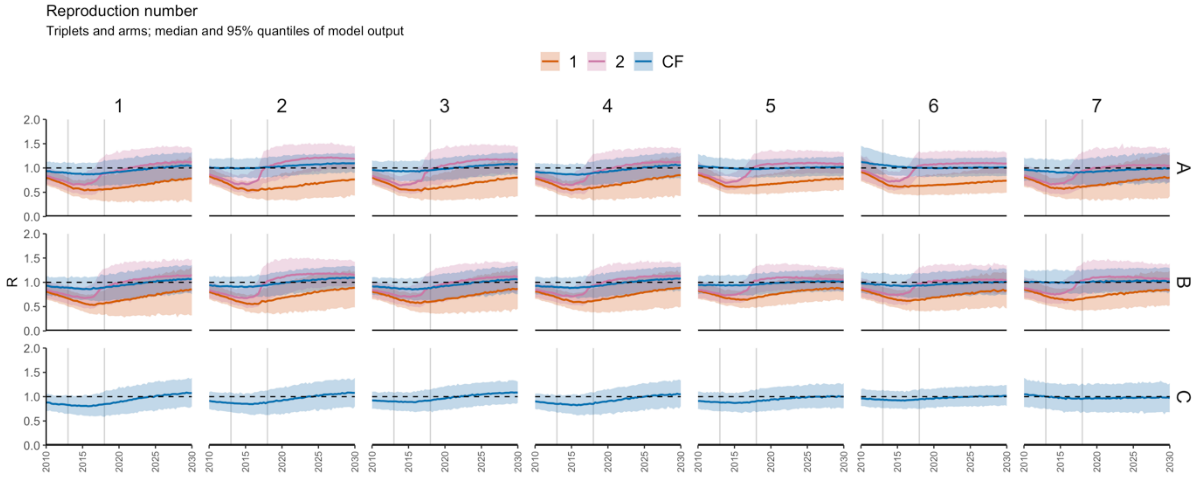

Figure S16: Median reproduction number (R) from 2010 to 2030 under three modelled scenarios (scenario 1, 2, counterfactual (CF)). Scenario 1 is continuation of PopART intervention, scenario 2 is discontinuation of the PopART intervention, counterfactual is a simulation of standard-of-care. Shaded area represents 95% credible intervals of model output over 1000 model parameter sets. Black dotted line is R=1.0. Grey vertical lines are the approximate start and end of the HPTN 071 (PopART) trial (2014, 2018).

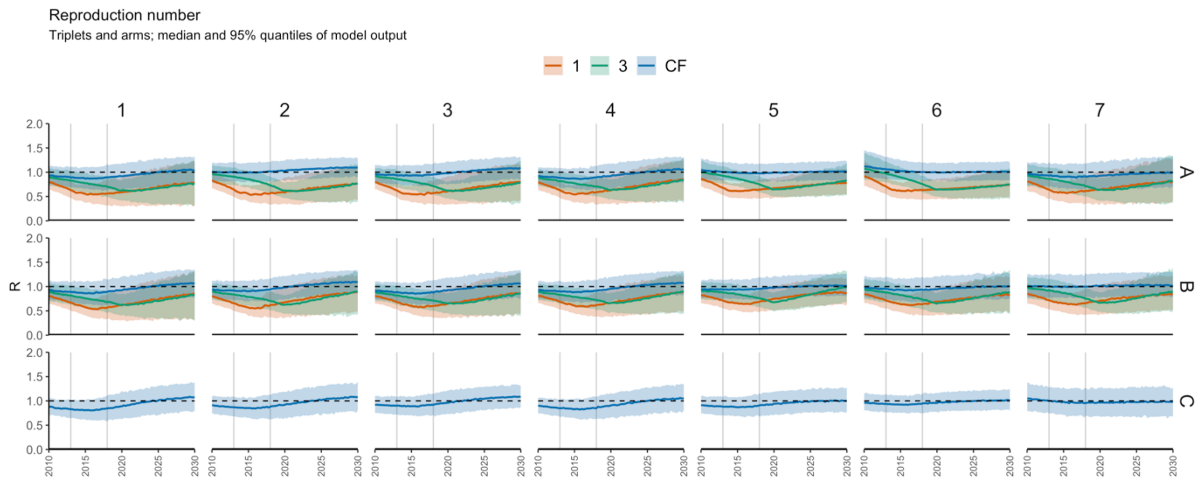

Figure S17: Median reproduction number (R) from 2010 to 2030 under three modelled scenarios (scenario 1, 3, counterfactual (CF)). Shaded area represents 95% credible intervals over 1000 model parameter sets. Black dotted line is R=1.0. Grey vertical lines are the approximate start and end of the HPTN 071 (PopART) trial (2014, 2018).

Voluntary medical male circumcision (VMMC)

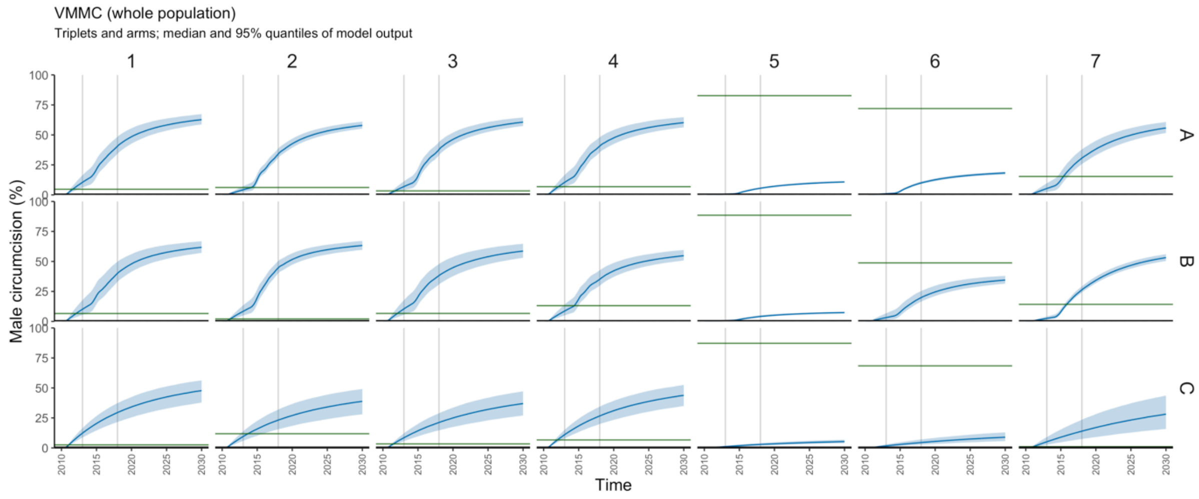

Figure S18: Scale up of VMMC in the model. Lines show median output with 95% credible intervals in shaded region. Dark green line shows percent of population assumed to have traditional male circumcision (and therefore not eligible for VMMC), also shown in table S4, below.

Proportion of traditional male circumcision	Community	Arm
0.0659	1	B
0.0468	2	A
0.025	3	C
0.1183	4	C
0.0618	5	A
0.0203	6	B

0.0334	7	C
0.0334	8	A
0.0666	9	B
0.0682	10	A
0.1312	11	B
0.0665	12	C
0.885	13	B
0.8271	14	A
0.8721	15	C
0.7195	16	A
0.6842	17	C
0.4876	18	B
0.1537	19	A
0.1421	20	B
0.0103	21	C

Table S4: Proportions of traditional male circumcision used in the modelling analyses.

Loss-to-follow-up/Dropout and treatment failure

Loss-to-follow-up / drop-out

The model simulated individuals dropping out of treatment. The mechanisms of drop-out are detailed in the paper describing the model (Pickles et al., 2021). Not modelling loss-to-follow-up may underestimate the prevalence of detectable viraemia (community viral burden), leading to underestimating the number of incident cases, particularly in intervention communities, and therefore lead to an overestimation of the intervention on reducing HIV incidence in intervention communities compared to arm C communities. It could also be argued that the rate of default from ART could be lower in intervention communities because of repeated visits of CHiPs to HIV-positive clients to provide treatment support. For a single calibrated model run, the annual dropout numbers, as a percentage of all those on ART are 2.36, 2.25, 2.44, 2.23, 2.11 over the years 2013-2017 respectively, which is similar to other studies (e.g. Granich et al., 2012).

Treatment failure

Treatment failure due to poor adherence is taken into account through loss-to-follow-up (described above) and through the explicit modelling of those who are on ART but not completely virally suppressed. For a calibrated parameter set from an intervention arm, the proportion of individuals assumed to be virally unsuppressed due to poor adherence or treatment failure ranges between 5.13% and 6.06% over the years 2013-2017 (trial period).

Insight into the dissonant triplets

A dissonant triplet is where HIV incidence was higher in arm A than arm C during PC12-36 (an unexpected result). Using the post-unblinding model, the model predicted one out of three of the dissonant triplets (triplet 5 out of triplets 1, 2, and 5; figure 3, S5; Hayes et al., 2019). In

triplet 5, high HIV incidence in arm A was driven by high HIV incidence in men (figure 3). Despite fitting the relative change in HIV incidence in almost all communities in triplets 1 and 2 when split by sex, the model projections did not capture, among any calibrated parameter sets, the unexpectedly high HIV incidence in women in arm A and men in arm A in triplets 1 and 2 respectively. There was no systematic difference in fit to the observed data indicating reasons for these subgroups to diverge in predictions of HIV incidence (figures S26.1-S26.6). In triplet 5, where the model did predict the dissonant triplet, the higher HIV incidence in arm A compared to arm C was due to differences in parameters governing engagement in the background care cascade between these two communities (i.e. the annual probability of women having an HIV test in the background care cascade and the probability of an individual picking up their CD4 test results; figures S27.7, S27.13). Estimates of these parameters were, in turn, driven by differences in the proportion of individuals amongst people living with HIV who were aware of their HIV status at trial baseline in arm C and A communities, where such awareness in arm A communities was lower across all age groups compared to arms B and C (figure S25). Awareness of HIV status is modelled via testing, and testing is the entry point to the care cascade (or via advanced HIV disease leading to emergency ART). Proportions of individuals aware of their HIV status at trial baseline were similar across all arms in triplets 1 and 2 (figure S25).

Outcomes in modelled scenarios, 2010-2030

For all scenarios, HIV incidence was estimated in the PopART community (i.e. the "inside" patch), despite the PopART intervention sometimes being simulated in the surrounding area (i.e. the "outside" patch). Where continuation of the PopART intervention was modelled after the trial it was assumed that the PopART intervention was delivered annually with the same final testing coverage (stratified by age and sex) as was observed in the final round of the trial.

Overall, counterfactual simulations produced similar projections of engagement with the HIV care cascade when compared to arm C simulations (tables S8-S10; scenario CF, arms A and B compared to arm C). Projections of awareness (of HIV+ status) were higher when fit to arm C compared to counterfactual simulations (table S8).

HIV incidence

Scenario	Men	Women	Total	Year
1	0.3789 (0.3201, 0.4481)	0.6757 (0.5775, 0.7802)	0.5208 (0.4469, 0.6055)	2020
2	0.4915 (0.4175, 0.58)	0.8248 (0.7106, 0.9533)	0.6515 (0.5638, 0.753)	2020
3	0.8057 (0.6925, 0.9303)	1.2229 (1.0627, 1.3936)	1.0046 (0.8833, 1.1356)	2020
CF	0.844 (0.7308, 0.9811)	1.2727 (1.0911, 1.4488)	1.0484 (0.9193, 1.187)	2020

Table S5: Projected mean annual HIV incidence in four modelled scenarios averaged across all intervention communities in 2020 (median, 95% CrI over 1000 parameter sets).

Scenario	Men	Women	Total	Year
1	0.2748 (0.2208, 0.3396)	0.4742 (0.3818, 0.5741)	0.3694 (0.3017, 0.4469)	2029
2	0.617 (0.522, 0.7348)	0.9292 (0.7782, 1.0846)	0.7655 (0.6527, 0.8861)	2029
3	0.2266 (0.184, 0.2815)	0.4038 (0.3293, 0.486)	0.3119 (0.2576, 0.3745)	2029
CF	0.7085 (0.6072, 0.8256)	1.0586 (0.909, 1.2229)	0.8746 (0.7542, 1.0083)	2029

Table S6: Projected mean annual HIV incidence in four modelled scenarios averaged across all intervention communities in 2029 (median, 95% CrI over 1000 parameter sets).

Scenario	Men	Women	Total	Year
1	0.2715 (0.2168, 0.3337)	0.4672 (0.3703, 0.5633)	0.3642 (0.2959, 0.4393)	2030
2	0.6196 (0.5239, 0.7344)	0.9309 (0.7832, 1.0886)	0.7673 (0.6568, 0.8927)	2030
3	0.2152 (0.1712, 0.2643)	0.3785 (0.3098, 0.4582)	0.2943 (0.2401, 0.3549)	2030
CF	0.6996 (0.5991, 0.8272)	1.0422 (0.8904, 1.2076)	0.8634 (0.7475, 0.9956)	2030

Table S7: Projected mean HIV incidence in four modelled scenarios averaged across all intervention communities in 2030 (median, 95% CrI over 1000 parameter sets).

Scenario	Arm	1	2	3	4	5	6	7
1	A	97.5 (95.52, 98.9)	96.87 (95.33, 98.31)	97.2 (95.46, 98.64)	97.37 (95.48, 98.86)	94.67 (93.44, 95.99)	95.29 (93.87, 96.47)	95.79 (93.78, 97.88)
1	B	97.68 (95.25, 99.2)	97.15 (94.97, 98.85)	97.16 (94.88, 98.82)	96.92 (94.68, 98.55)	94.38 (92.96, 95.98)	95.49 (93.48, 97.59)	93.98 (92.35, 95.84)
2	A	85.62 (75.29, 92.98)	72.96 (66.06, 81.38)	80.55 (71.17, 88.6)	86.3 (78.17, 92.97)	71.52 (65.93, 77.88)	70 (62.97, 75.84)	81.2 (71.92, 89.59)
2	B	86.24 (76.81, 94.08)	81.96 (74.54, 89.62)	86.81 (77.36, 94.21)	85.13 (76.93, 91.83)	78.18 (70.88, 85.77)	80.36 (70.37, 90.32)	71.88 (67.68, 77.4)
3	A	97.37 (95.74, 98.58)	96.97 (95.62, 98.04)	97.22 (95.75, 98.38)	97.39 (95.8, 98.63)	94.66 (93.15, 95.96)	95.32 (93.86, 96.44)	95.65 (93.67, 97.53)
3	B	97.62 (95.6, 98.86)	97.37 (95.52, 98.69)	97 (94.93, 98.58)	96.98 (94.86, 98.38)	94.51 (92.91, 96.19)	95.48 (93.37, 97.53)	94 (92.39, 95.54)
CF	A	83.51 (71.88, 91.48)	65.98 (57.29, 75.51)	76.31 (64.24, 85.35)	84.79 (75.84, 91.93)	63.4 (56.76, 72.89)	60.75 (53.8, 68.83)	77.23 (64.5, 87.26)
CF	B	84.36 (72.54, 93.15)	79.21 (69.87, 87.82)	85.1 (74.03, 92.95)	83.33 (73.77, 90.66)	74.71 (63.54, 84.98)	76.6 (61.79, 88.79)	64.12 (58.62, 71.16)
CF	C	88.64 (83.61, 92.72)	85.92 (77.45, 92.02)	81.35 (74.68, 87.39)	86.96 (81.71, 92.06)	83.61 (77.89, 88.07)	75.06 (67.49, 82.56)	70.67 (59.29, 81.65)

Table S8: Projected mean proportion of individuals aware of HIV status (1st 90) by 2030 amongst people living with HIV under four scenarios of universal testing and treatment in each intervention community of the trial (columns labelled 1-7 are triplet number). Projections are for the PopART community so not including the surrounding area. Values are median across 1000 parameter values, brackets show 95% credible intervals.

Scenario	Arm	1	2	3	4	5	6	7
1	A	96.23 (93.96, 97.91)	95.84 (93.76, 97.51)	96.33 (94.23, 97.9)	96.28 (94.18, 98)	94.22 (91.42, 95.95)	93.47 (90.27, 95.79)	95.38 (93.07, 97.08)
1	B	96.66 (94.61, 98.25)	96.59 (94.9, 98.07)	96 (93.51, 97.71)	96.04 (93.73, 97.59)	95.1 (93.69, 96.26)	94.59 (91.52, 96.7)	94.61 (92.48, 96.16)
2	A	90.66 (86.97, 93.51)	91.53 (88.01, 94.06)	91.54 (87.92, 93.9)	90.86 (86.87, 93.58)	91.35 (87.4, 93.81)	89.21 (84.32, 93.06)	91.9 (88.59, 94.15)
2	B	91.1 (87.43, 93.63)	91.79 (89.03, 93.67)	90.61 (86.54, 93.18)	90.73 (86.63, 93.11)	92.85 (90.84, 94.33)	90.45 (85.94, 93.64)	92.62 (90.1, 94.39)
3	A	94.97 (92.42, 96.85)	94.61 (91.81, 96.39)	95.1 (92.37, 96.77)	95.24 (92.66, 97)	92.81 (89.51, 94.9)	91.75 (87.97, 94.68)	94.1 (91.35, 95.93)
3	B	95.66 (93.06, 97.3)	95.93 (94.19, 97.32)	94.88 (92.1, 96.79)	95.17 (92.47, 96.85)	94.45 (92.83, 95.74)	93.48 (89.77, 95.7)	93.44 (90.76, 95.1)
CF	A	87.88 (84.02, 90.77)	89.32 (85.67, 91.99)	88.82 (85.33, 91.29)	88.05 (84.11, 90.79)	89.46 (85.66, 92.33)	86.79 (81.8, 91.22)	89.71 (86.25, 92.38)
CF	B	88.55 (84.92, 91.05)	89.31 (86.85, 91.25)	87.97 (83.99, 90.74)	88.2 (84.43, 90.72)	91.26 (89.13, 93.28)	88.27 (83.94, 91.81)	91.4 (89, 93.38)
CF	C	90 (87.93, 91.52)	89.63 (86.91, 91.48)	89.54 (87.07, 91.4)	89.11 (86, 91.21)	90.53 (88.22, 92.05)	89.52 (85.64, 92.3)	87.04 (81.7, 91.34)

Table S9: Projected mean proportion of individuals on ART (2nd 90) amongst people aware of HIV status by 2030 under four scenarios of universal testing and treatment in each intervention community of the trial (columns labelled 1-7 are triplet number). Projections are for the PopART community so not including the surrounding area. Values are median across 1000 parameter values with arithmetic mean taken across intervention communities, brackets show 95% credible intervals.

Scenario	Arm	1	2	3	4	5	6	7
1	A	98.13 (97.26, 98.91)	97.84 (96.9, 98.6)	98.09 (97.26, 98.76)	98.13 (97.2, 98.84)	97.47 (96.54, 98.12)	97.15 (96.04, 98.01)	98 (97.26, 98.66)
1	B	98.22 (97.33, 98.95)	98.08 (97.31, 98.76)	98.09 (97.18, 98.81)	97.96 (97.02, 98.68)	97.76 (97.24, 98.22)	97.66 (96.67, 98.43)	97.65 (96.98, 98.27)
2	A	98.09 (97.41, 98.78)	98.23 (97.62, 98.7)	98.2 (97.6, 98.72)	98.07 (97.39, 98.71)	98.28 (97.66, 98.71)	98.12 (97.26, 98.7)	98.32 (97.73, 98.79)
2	B	98.13 (97.47, 98.8)	98.11 (97.58, 98.69)	98.09 (97.36, 98.67)	97.98 (97.31, 98.59)	98.26 (97.86, 98.66)	98.14 (97.5, 98.63)	98.39 (97.9, 98.8)
3	A	97.52 (96.67, 98.27)	97 (96.03, 97.74)	97.36 (96.45, 98.05)	97.54 (96.66, 98.28)	96.63 (95.71, 97.31)	96.24 (95.05, 97.1)	97.34 (96.55, 98)
3	B	97.67 (96.73, 98.4)	97.58 (96.85, 98.25)	97.53 (96.61, 98.3)	97.48 (96.53, 98.17)	97.27 (96.67, 97.86)	97.06 (95.99, 97.89)	96.88 (96.08, 97.52)
CF	A	97.34 (96.65, 98.09)	97 (96.4, 97.5)	97.19 (96.59, 97.78)	97.38 (96.7, 98.04)	97.16 (96.46, 97.72)	96.78 (95.82, 97.52)	97.51 (96.88, 98.04)
CF	B	97.43 (96.75, 98.19)	97.29 (96.8, 97.9)	97.43 (96.71, 98.15)	97.32 (96.65, 97.94)	97.58 (97.12, 97.97)	97.36 (96.65, 97.94)	97.45 (96.78, 97.97)
CF	C	97.73 (97.2, 98.25)	97.59 (97, 98.16)	97.37 (96.89, 97.95)	97.6 (97.01, 98.18)	97.67 (97.18, 98.03)	97.41 (96.79, 97.9)	96.98 (95.98, 97.78)

Table S10: Projected mean proportion of individuals viral suppressed (3rd 90) amongst people on ART by 2030 under four scenarios of universal testing and treatment in each intervention community of the trial (columns labelled 1-7 are triplet number). Projections are for the PopART community so not including the surrounding area. Values are median across 1000 parameter values with arithmetic mean taken across intervention communities, brackets show 95% credible intervals.

Inference framework and parameterisation

The inference framework uses Approximate Bayesian Computation (ABC) to fit the model to several sources of observed data. Further to the details provided in the main text and also in Pickles et al., (2021), this document describes: 1) timings of the CHiPs (intervention), Population Cohort (PC), and population survey (DHS and HSRC) rounds as used in the model, 2) the CHiPs testing coverage used in the model, 3) the statistical model of viral suppression used to generate data for fitting, 4) the parameters varied in the inference framework, 5) the unblinding process, 6) outputs from the inference framework including model fit to the observed data and parameter posterior distributions.

Period/Round	Country	Start date	End date
Round 1	Zambia	1 November 2013	30 June 2015
Round 2	Zambia	1 June 2015	23 October 2016
Round 3	Zambia	1 September 2016	31 December 2017
Round 1	South Africa	1 January 2014	30 April 2015
Round 2	South Africa	1 June 2015	5 August 2016
Round 3	South Africa	1 September 2016	31 December 2017
Post-trial rounds	Zambia and South Africa	1 January, year in question	31 December, year in question

Table S11: Timings of rounds of the PoPART intervention. Dates shown are the limits within each country, community-specific start and end dates vary slightly from these values as community-specific CHiPs testing schedules are used in the model. "Post-trial" rounds show dates over which the simulated intervention is performed when the scenarios simulate the PopART intervention after the end of the trial (e.g. in the modelled scenarios).

Survey	Start date	End date	Source
HSRC 2002	1 May 2002	30 September 2002	HSRC 2002
HSRC 2005	1 October 2004	30 June 2005	HSRC 2005
HSRC 2008	1 May 2008	31 March 2009	HSRC 2008a, 2008b
HSRC 2012	1 August 2011	31 October 2012	HSRC 2012
DHS 2002	November 2001	May 2002	Central Statistical Office/Zambia 2003
DHS 2007	April 2007	November 2007	Central Statistical Office/Zambia 2009
DHS 2013	August 2013	April 2014	Central Statistical Office/Zambia 2014

Table S12: Timings of rounds of the historical prevalence surveys from DHS and HSRC. Within the model, the year of the survey (in the survey name) was used and not the exact dates.

CHiPs testing coverage

Coverage of household testing by CHiPs teams was estimated from trial data and stratified by year of age and gender of the person receiving the CHiPs visit. These schedules were used as model inputs for each intervention community. Such data were not available for South African communities for CHiPs rounds 1 and 2 and so CHiPs testing schedules for these two rounds were generated by scaling the South African round 3 data such that the final coverage for each age and gender in round 2 or 1 is the same as the respective change overall in the Zambian communities receiving the PopART intervention. For instance, the age/gender scaling for the CHiPs testing coverage from round 3 to 2 in Zambia is used to generate round 2 CHiPs testing coverage in South Africa from the round 3 testing coverage in South Africa.

The model assumes that, at a CHiP-visit level, the PopART intervention was equally efficacious across communities, and that variability in delivery of the PopART intervention is accounted for by differences in CHiPs coverage (as presented in figure S19-S22; SoC efficacy can still vary across communities). Any differences in service delivery of CHiPs will therefore be accounted for during the model fitting process via other mechanisms in the model, such as the estimated efficacy of the background care cascade. Relaxing this assumption leads to issues of model identifiability. This will have an impact on counterfactual simulations and may explain why, before data unblinding, intervention impact in arm A is estimated as more effective than arm B communities (intervention impact before data unblinding was calculated against counterfactual simulations). However, this limitation will not affect projections that include both the PopART intervention and the background care cascade together. During the trial, regular monitoring of CHiP data was carried out (Floyd et al., 2020).

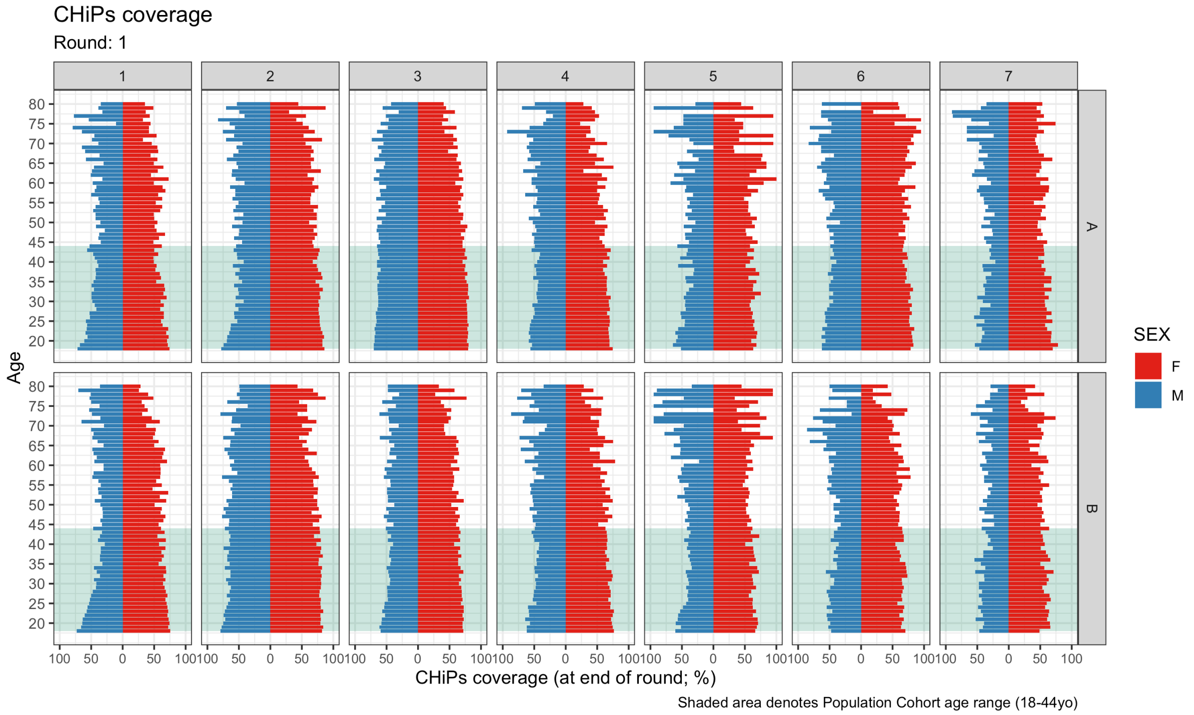

Figure S19: CHiPs coverage stratified by age and sex in all intervention communities of HPTN 071 (PopART) as used in modelling inputs for round 1.

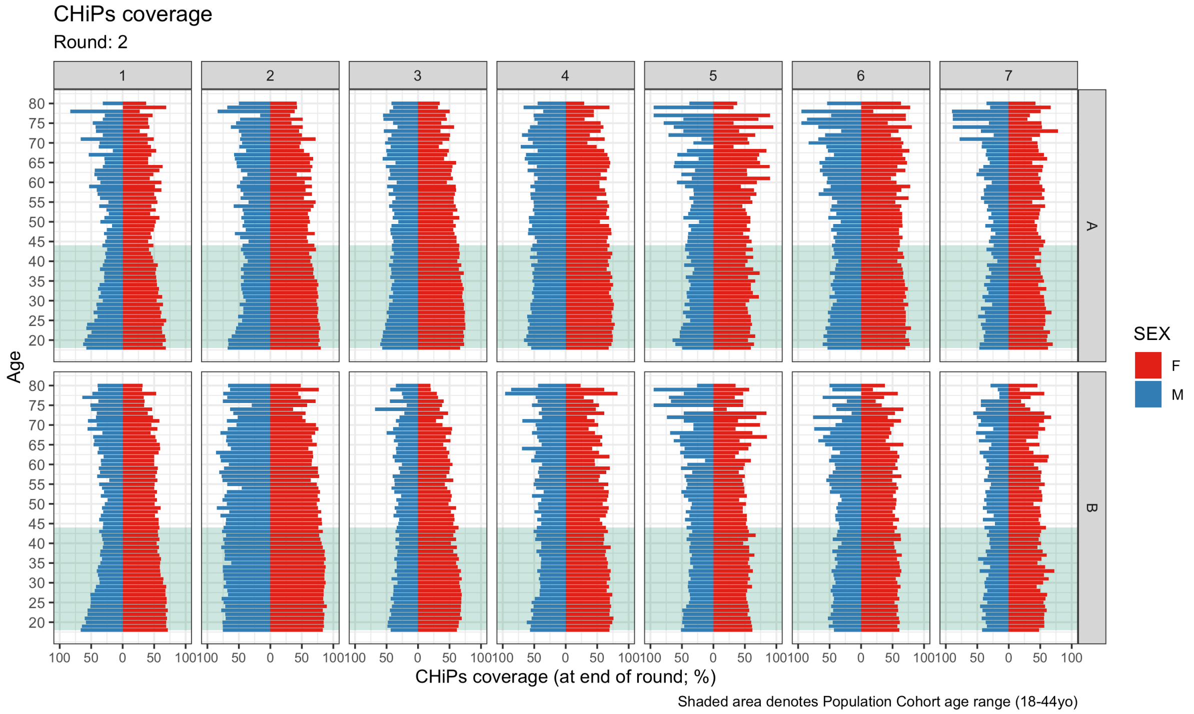

Figure S20: CHiPs coverage stratified by age and sex in all intervention communities of HPTN 071 (PopART) as used in modelling inputs for round 2.

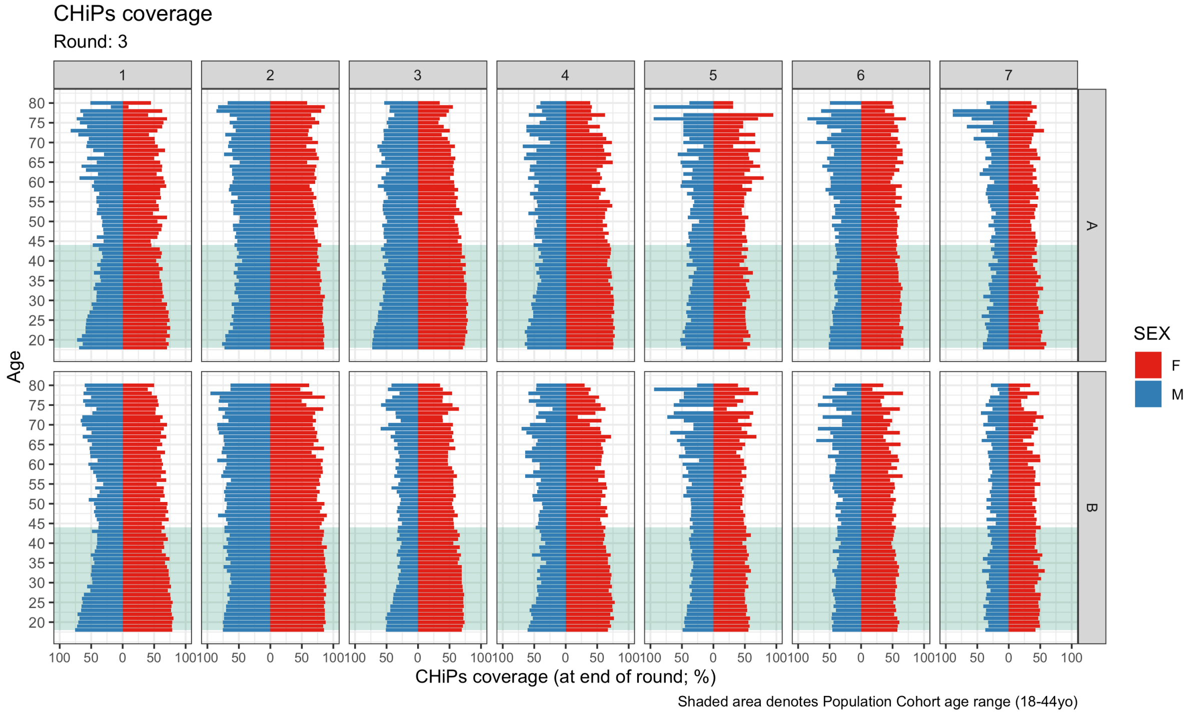

Figure S21: CHiPs coverage stratified by age and sex in all intervention communities of HPTN 071 (PopART) as used in modelling inputs for round 3.

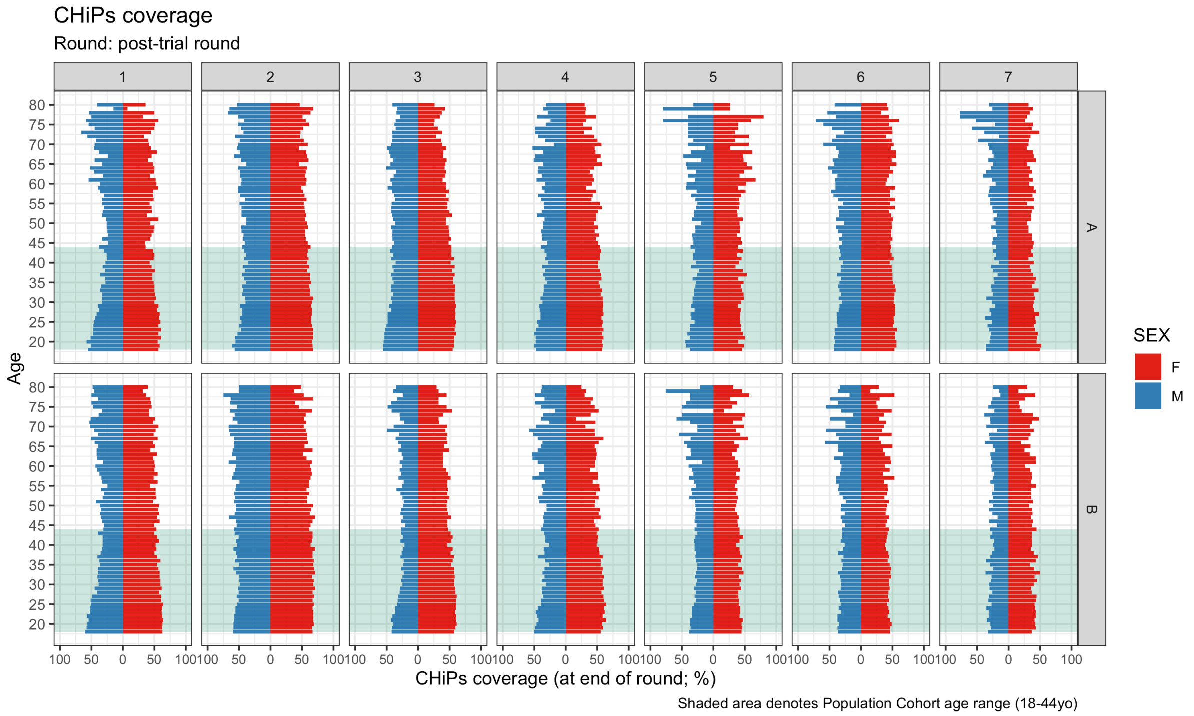

Figure S22: CHiPs coverage stratified by age and sex in all intervention communities of HPTN 071 (PopART) as used in modelling inputs for post-trial rounds.

Statistical model of viral suppression

The number of virally suppressed HIV positive trial participants at PC24 in each trial community by age-group and sex was small which results in large variance and heterogeneity of the estimated proportions of virally suppressed individuals among HIV positives in each stratum.

The individual-based model structure and assumptions do not allow mimicking of these random fluctuations between strata. It would have not been meaningful to improve the fit to the proportion of virally suppressed which also implies a tradeoff with minimizing the distance for remaining summary statistics (prevalence, proportion on ART). Therefore, a statistical regression model with additional assumptions was used to generate predictions of the proportion of virally suppressed in each community by age-groups and sex at PC24. Predicted proportions of virally suppressed from this statistical model were used to calibrate model projections, instead of crude proportions.

The statistical regression included a factor for trial communities, one for sex, and one for the PC rounds (PC0, PC12, PC24). A random walk model of order one was assumed for neighboring age-groups, irrespective of community and sex. This implies that information is lent across communities and sex to shrink the estimated proportion of virally suppressed towards the overall mean of a particular age-group. The amount of shrinkage is determined by the estimated autoregression coefficient for neighboring age-groups.

The regression model was defined as a Bayesian hierarchical model and estimated with integrated nested Laplace approximation via the R-package INLA (Rue et al., 2009). The following prior distributions on the model parameters were used: the regression coefficient for the sex-factor was assumed to follow a normal distribution with mean zero and a variance of 1000. A Gamma distribution with shape parameter $\alpha = 0.5$ and scale parameter $\beta = 0.0005$ was used for the precision of the community and PC-round factors. The random walk model

of order one for the 5-year age brackets assumed that these are grouped and exchangeable across communities and sex. Values were scaled such that the variance of the random walk model was equal to one. A penalized complexity prior (Simpson et al., 2017) with parameters u = 1 and α = 0.01 was assigned to the precision of the random-walk of order one.

Inference framework

Wihtin this study, the adaptive Population Monte Carlo ABC algorithm (PMC-ABC) in Lenormand et al. (2015) was used with an acceptance parameter of 5%. The number of complete simulations run by the ABC varied by community (between 40,000 and 130,000) and the framework returned 1000 calibrated parameter sets that were used in subsequent analyses.

Parameter description	Lower limit	Upper limit	Distribution
Risk assortativity	0.05	0.95	Uniform
Baseline probability of a woman having an HIV test in the period before 2006.	0.1	0.2	Uniform
Baseline annual probability of a woman having an HIV test in a given year after 2006	0.05	0.4	Uniform
Multiplier for baseline annual probability of a man having an HIV test in a given year compared to a woman.	0.4	1.1	Uniform
Annual hazard of transmission from an HIV+ individual to a HIV- partner.	0.05	0.3	Uniform
Probability that an individual not in PopART collects CD4 test results and therefore joins pre-ART care.	0.75	0.95	Uniform
Multiplier of hazard of HIV infection if transmission is from male to female.	1	3	Uniform
Proportion of men in the population that are in low risk sexual activity class on entry to the model	0.3	0.6	Uniform
Proportion of women in the population that are in low risk sexual activity class on entry to the model	0.3	0.6	Uniform
Proportion of men in the medium risk activity class compared to the high sexual risk activity class on entry to the model	0.5	0.99	Uniform
Proportion of women in the medium risk activity class compared to the high sexual risk activity class on entry to the model	0.5	0.99	Uniform
Multiplier to account for potential over/under-reporting of number of sexual partners.	0.625	5	Uniform
Log of the multiplier for the initial proportion of individuals that are infected (stratified by age and sex) during the HIV seeding process.	0	2	Uniform
Mean time delay (in years), as drawn from an exponential distribution, until starting ART after testing HIV+ for an individual in the background care cascade.	0.4	0.7	Uniform

Multiplier to scale parameter for gamma distribution determining the duration of partnerships within a patch.	1	2	Uniform
Probability that a woman stays virally suppressed after the initial ART phase	0.65	0.9	Uniform
Multiplier for the probability that a man stays virally suppressed after the initial ART phase compared to a women	0.6	1	Uniform

Table S13: Parameters estimated in the calibration framework in the post-unblinding model. Uniform priors were used for all parameters with ranges shown in the table.

Fixed parameters are provided in the zipped data files included in the repository.

No weighting was used for each of the summary statistics used in the fitting process although it is noted that weighting by sample size or uncertainty bounds of each of the respective datasets could have been used.

Unblinding process

Projections of intervention impact and selected secondary endpoints were made at several points throughout the trial. Projections in 2014 (Cori et al., 2014) were made with a deterministic, compartmental model, all other projections used a version of the model presented in this analysis and documented in detail in Pickles et al., 2021. Projections from Cori et al., 2014 suggested a 3-year reduction in HIV incidence of 61% and 62% for arm A, and 25% and 26% in arm B communities in Zambia and South Africa respectively (assuming 6-month CHiPs rounds). Projections in Oct 2016, Nov 2017, and Jan 2018 were provided as three reports given to an external data safety and monitoring board (DSMB; sometimes called "data monitoring committees"), a committee of experts external to the trial. Further details of the DSMB are provided in section 8.8.2 of the trial protocol; HPTN 071 (PopART) Trial Protocol V3.0 (2015) and, in general, in Ellenberg et al., (2007). Specifically, projections were presented to the DSMB in October 2016 (for arm A communities in Zambia), to the DSMB in November 2017 (for all arm A communities), to the DSMB in January 2018 (for all arm B communities).

Before 17th December 2018, data collection for all PC rounds was complete but trial results were not unblinded to trial investigators (the modelling team was still blinded to all but the PC0 data). Pre-unblinding projections of the primary endpoint and several secondary endpoints were lodged in an online time-stamped data repository and in a sealed physical envelope. These activities were carried out by 4pm on 17th December 2018 (GMT) and the realised primary endpoint of the trial was reported to a selection of investigators at 9am on 17th December in Seattle, Washington, USA (GMT – 8).

Figure S23 provides a timeline of when projections were made for different communities. Figure S24 provides a summary of the different projections of intervention impact (the deterministic model is excluded from figure S24).

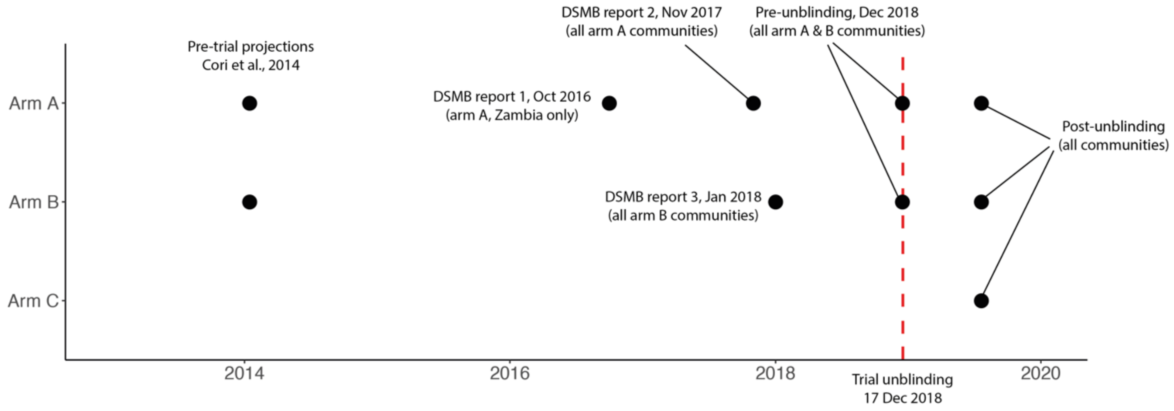

Figure S23: Timeline of different projections of the PopART intervention impact.

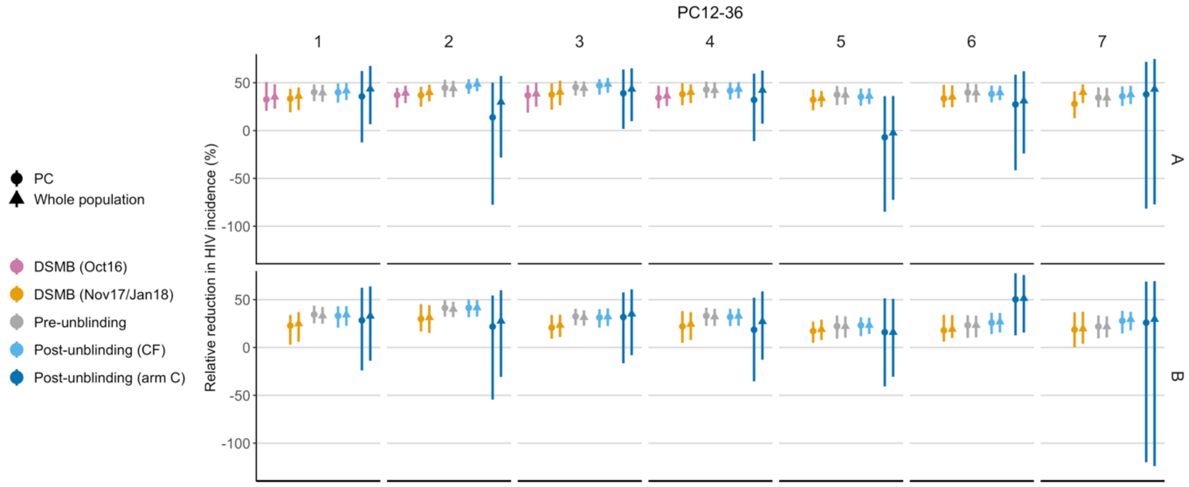

Figure S24: Projections of intervention impact at different points throughout the trial. Refer to figure S23 (above) for dates when the different projections were made (DSMB on Nov. 17 and Jan. 18 Projections are included together). All comparisons are against counterfactual simulations except post-unblinding projections denoted “arm C”.

Before data unblinding, 15 parameters were calibrated for each arm A or B community according to the following data (stratified by age and sex): 1) HIV prevalence from historical surveys (three DHS rounds in Zambia and four HSRC rounds in South Africa); 2) HIV prevalence from the final CHiPs round, 3) the proportion of HIV-positive individuals aware of their status for three rounds of CHiPs data (Zambia) and the final round of CHiPs data (South Africa), and 4) the proportion of individuals on ART amongst those aware of their HIV-positive status for three rounds of CHiPs data (Zambia) and the third round of CHiPs data (South Africa).

After data unblinding, in addition to the data used for calibration before December 2018, the model was calibrated to additional data (also stratified by age and sex) : 5) HIV prevalence from four rounds of the PC data, 6) viral suppression at 24 months after trial start (PC24), 7) the proportion of HIV-positive individuals aware of their status from four rounds of PC data in South Africa, and 8) the proportion of individuals on ART amongst those aware of their HIV-positive status from four rounds of PC data in South Africa.

Data source	Time	HIV prevalence	Proportion aware of HIV status (among PWH)	Proportion on ART (among those aware of HIV status)	Proportion virally suppressed (among PHW)

PC	PC0	O			
	PC12	X			
	PC24	X			X
	PC36	X			
CHiPs	R1		O	O	
	R2		O	O	
	R3	O	O	O	
DHS	2002	O			
	2007	O			
	2013	O			

Table S14: Data used as part of the fitting process in Zambian intervention communities. Circles (O) represent data that was used throughout the trial, crosses (X) represent data that was used only after trial unblinding.

Data source	Time	HIV prevalence	Proportion aware of HIV status (among PWH)	Proportion on ART (among those aware of HIV status)	Proportion virally suppressed (among PWH)
PC	PC0	O	X	X	
	PC12	X	X	X	
	PC24	X	X	X	X
	PC36	X	X	X	
CHiPs	R1				
	R2				
	R3	O	O	O	
HSRC	2002	O			

	2005	O			
	2008	O			
	2012	O			

Table S15: Data used as part of the fitting process in South African intervention communities. Circles (O) represent data that was used throughout the trial, crosses (X) represent data that was used only after trial unblinding.

Data source	Time	HIV prevalence	Proportion aware of HIV status (among PWH)	Proportion on ART (among those aware of HIV status)	Proportion virally suppressed (among PWH)
PC	PC0	X	X	X	
	PC12	X	X	X	
	PC24	X	X	X	
	PC36	X	X	X	X
CHiPs	R1				
	R2				
	R3				
DHS	2002	X			
	2007	X			
	2013	X			

Table S16: Data used as part of the fitting process in Zambian arm C communities. Circles (O) represent data that was used throughout the trial, crosses (X) represent data that was used only after trial unblinding.

Data source	Time	HIV prevalence	Proportion aware of HIV status (among PWH)	Proportion on ART (among those aware of HIV status)	Proportion virally suppressed (among PWH)
PC	PC0	X	X	X	
	PC12	X	X	X	

	PC24	X	X	X	X
	PC36	X	X	X	
CHiPs	R1				
	R2				
	R3				
HSRC	2002	X			
	2005	X			
	2008	X			
	2012	X			

Table S17: Data used as part of the fitting process in South Africa arm C communities. Circles (O) represent data that was used throughout the trial, crosses (X) represent data that was used only after trial unblinding.

Before trial unblinding, the prevalence of traditional male circumcision (TMC) was fixed at 9.43% of the male population in Zambia and 56.08% in South Africa, based upon analysis of PC0 data. After trial unblinding, the average prevalence of TMC observed across the PC rounds was used for each community (Table S4; country-level averages were still similar to the prevalence in the pre-unblinding parameters).

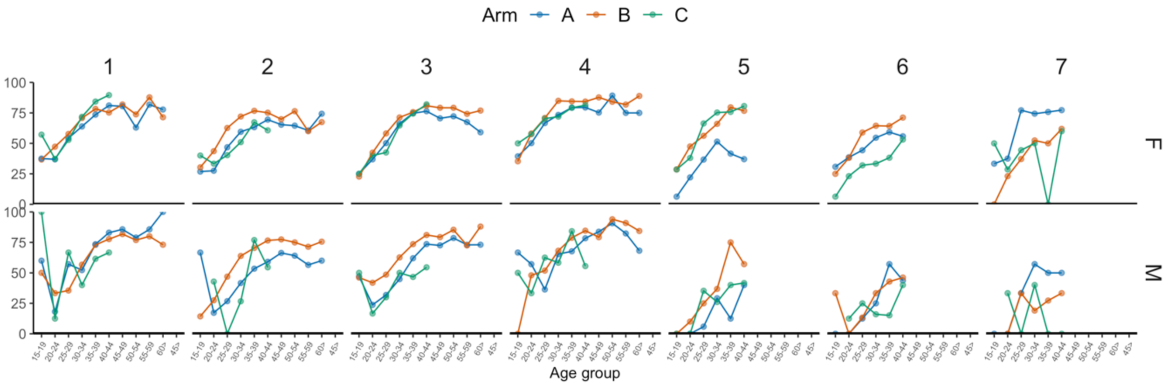

Figure S25: Proportion aware of their HIV status among people living with HIV prior to round 1 (PC0 in all South African communities or arm C in Zambia, or CHiPs R1 in intervention communities in Zambia).

Model fit

Figures S26.1-S26.21 present the model fit to observed data, stratified by age and sex, for all 21 communities in Zambia and South Africa and are provided as a compressed folder in the below repository. Figures S26 are labelled according to the scheme S26.C.M.T for community

C (1-21), metric M (HIV, AWARE, ART, VS), and time T (PC0, PC12, etc). The metrics stand for HIV prevalence (HIV), proportion aware of HIV status (AWARE), proportion on ART (ART), and proportion virally suppressed (VS). For instance, figure S26.3.HIV.PC12 shows HIV prevalence in community 3 over PC12 time period.

Overall, the model provided a reasonable fit to HIV prevalence and the HIV care cascade in all 21 communities, disaggregated by age and sex and over multiple time points. The model was designed to simulate epidemics in communities with HIV prevalence greater than 10% and so struggled to estimate prevalence in triplet 7 where two communities reported prevalence of approximately 3% (Hayes et al., 2019; Figs S26.18 - S26.21).

These figures are provided in a zipped folder at https://github.com/BDI-pathogens/popart_pre_post_unblinding_figures because they are numerous.

Marginal posterior distributions of fitted parameters

Figures S27.1-S27.17 present the marginal posterior distributions of the 17 parameters used in the calibration process for all 21 communities for Zambia and South Africa in the post-unblinding fit of the model. Several estimated parameter posteriors were highly peaked (figures S27.1-S27.17).

These figures are provided in a zipped folder at https://github.com/BDI-pathogens/popart_pre_post_unblinding_figures because they are numerous.

Parameter correlations

Figures S28 presents bivariate plots of correlations between marginal posteriors of parameters for community 1 (arm B). Bivariate plots are included for correlations that have an R^2 value of over 0.3.

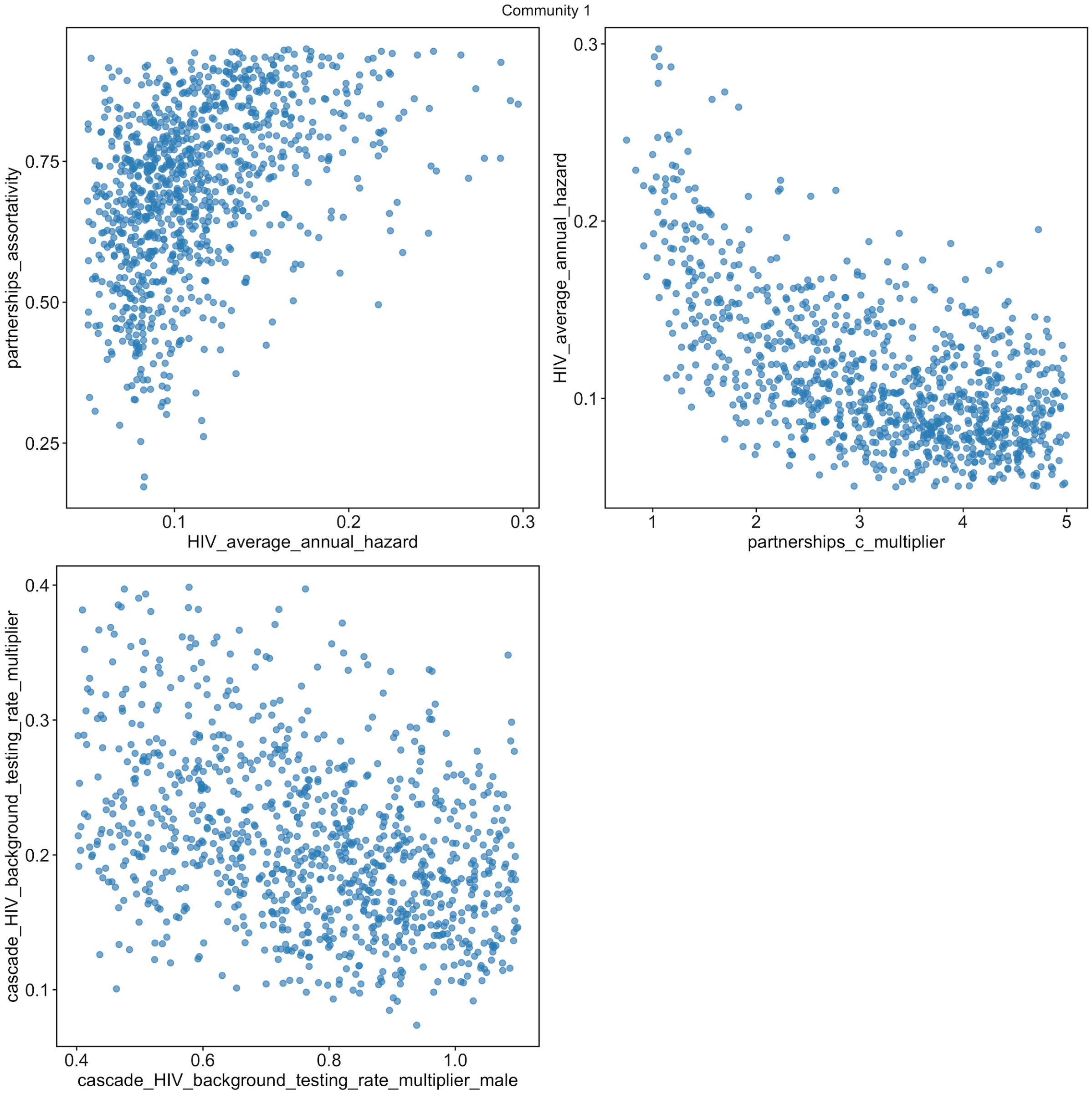

Figure S28: Bivariate scatterplot of marginal posterior distributions of parameters for community 1 with a Pearson's correlation over 0.3.

Code and analyses

Data processing, statistical analyses, and visualisation were performed in Python v3.8 and R v3.6.3. Code for the model is available at https://github.com/BDI-pathogens/POPART-IBM, code for pairing the model with the calibration framework and for post-processing results to produce figures and tables in this paper is provided upon request.

Code organisation and deployment pipelines

At a broad level, the PopART modelling team managed four types of code repositories: 1) the model codebase, 2) the codebase for data pre-processing (preparing parameters in the model that would be fixed and estimates of observed data used in the inference framework), 3) the codebase for the inference framework, 4) code for deploying computational experiments using the model in combination with parameter sets that had been generated using the inference framework (combining (2) and (3)). This is in line with viewing analyses with epidemiological models as a directed acyclic graph (DAG; figure S29) where output from one subsection of an analysis is input to another.

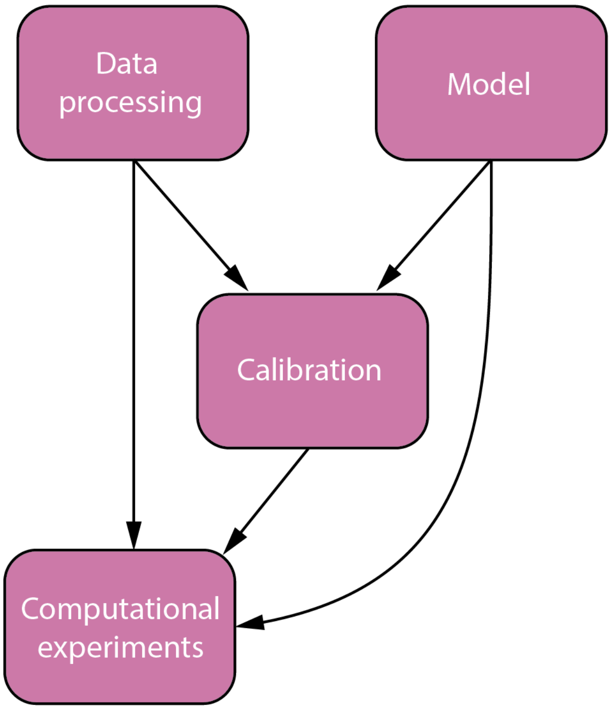

Figure S29: Organisation of code for PopART computational modelling analyses involving four types of repositories, phrased as a directed acyclic graph: 1) the model, 2) the codebase for data pre-processing, 3) the codebase for the inference/calibration framework, 4) codebase(s) for deploying computational experiments using the model in combination with calibrated parameter sets.

The code was partitioned in this manner for a number of reasons: 1) these bodies of code naturally progress at different times and for different reasons within the project, 2) testing of individual components within the DAG is manageable and compartmentalised, 3) this provides a natural separation of confidential and publicly available data (i.e. only the data processing repository needs to interact with confidential data), 3a) this separates code from data (that may be confidential), 4) external partners typically only need access to the fourth repository type (code for deploying computational experiments) so it removes the need for subsetting analyses when sharing analyses, 5) this allows different team members to work independently while minimizing conflicting contributions to the codebase, 6) any changes in the commit history of each repository then also records meaningful changes in that aspect of the analysis, 7) this structure reinforces the notion that a model is not an inference framework - models can be paired with several calibration frameworks and this allows “plug-and-play” approach of matching models with calibration frameworks (or other frameworks such as optimisation frameworks or for testing), 8) simplified deployment by only using the needed repositories (rather than a large repository with all code), 9) disentangling computational prerequisites needed to run different components of the model (i.e. the code calibration framework is not needed to run sensitivity analyses, for instance), 10) simplified versioning that was required during testing and development of each component.

In regard to (6), for example, imagine if all code is in one repo (model code and calibration code) then the commit history of the calibration framework and model is intertwined and developers will need to use their own versioning system to keep track of how things changed). For instance, if an error is found in the model and a patch is made to the model code to fix this then if the model and calibration are stored in separate repositories the commit history of the model only changes, the version of the calibration is still the same. If they’re in one repository the commit history of both is changed and one will need to keep track of the calibration separately. Benefits of a single repository (a monorepo) is that conflicts between different histories/version of different repositories is minimised. Larger, interconnected repositories may want to consider housing code in a monorepo.

Pipelines were primarily written using shell scripts or GNU Make, which is portable across Mac and most Linux operating systems. Tests for the model were written in Python 3.6 using the pytest framework. The tests are provided in the model repository.

Being agile and adaptable allows computational experiments to be deployed more quickly, sensible version control allows faster resolution of issues, and ultimately these all contribute to more efficient use of human resources.

Glossary

Term	Definition	Reference
Average HIV incidence rate	The average rate of new HIV infections over a specified period of time. Calculated as the ratio of new HIV infections over person-years of observation. Figure 3 presents model-predicted average HIV incidence rate for 1000 parameter sets. Units are typically ‘rate per 100 person-years’.	
Data Safety and Monitoring Board (DSMB)	An independent committee including expertise in HIV prevention, statistics, cluster-randomized trials and clinical medicine (including antiretroviral	HPTN 071 (PopART) Trial Protocol V3.0

	therapy). The responsibilities of the DSMB will be to monitor data from the trial and to advise the sponsor and study leadership on any recommended changes to the conduct of the study including early termination for futility on the primary endpoint if appropriate.	(2015); Ellenberg et al., 2007; Hayes and Moulton (2022)
Rate ratio (RR), incidence rate ratio (or risk ratio)	The ratio of two HIV incidence rates (or average HIV incidence rates, above). This is used to calculate primary endpoint of the trial (ratio of incidence rates in intervention arm and control arm respectively).	Lash, et al., (2021)
Relative reduction in HIV incidence	1 – RR (see above). Typically expressed as a percentage. This is the primary endpoint of the HPTN 071 (PopART) trial, figure 2 presents the relative reduction in HIV incidence.	
Population Cohort (PC)	The cohort within which the primary endpoint of the trial was measured. The Population Cohort was a random sample of approximately 2000 adults aged 18-44yo in each of the trial communities. This cohort was approximated within the model.	HPTN 071 (PopART) Trial Protocol V3.0 (2015)
CHiPs	Community HIV-care Providers ("CHiPs") were members of the communities of the trial that delivered the PopART intervention.	HPTN 071 (PopART) Trial Protocol V3.0 (2015)
PopART intervention	The intervention that was implemented as part of the HPTN 071 (PopART) trial.<br><br>The trial intervention was composed of annual rounds of home-based HIV testing, including support for linkage to care and retention on antiretroviral therapy (ART) at government primary health care facilities, promotion of medical male circumcision for HIV-negative men, and additional HIV, TB, and STI services, and was delivered by teams of Community HIV-care Providers ("CHiPs").	Hayes et al., (2019); HPTN 071 (PopART) Trial Protocol V3.0 (2015)
Unblinding	Referring to the data unblinding process of the trial, this is where investigators were blinded to the data that was measuring the primary endpoint of the trial. Data unblinding occurred on 18th December 2018.	HPTN 071 (PopART) Trial Protocol V3.0 (2015); Donnell et al., (2018) Hayes et al., (2019)
UTT	Universal testing and treatment is an HIV prevention intervention proposed in high HIV prevalence areas involving *offering HIV voluntary counselling and testing (VCT) to the entire population, and offering immediate ART to all those testing HIV-positive irrespective of clinical stage or CD4 count* (Hayes, Sabapathy, Fidler, 2011).	Montaner et al., (2006); Hayes, Sabapathy and Fidler (2011)

Note on terminology

The individual-based model used in this analysis (Pickles et al., 2021 simulates heterosexual transmission of HIV where risk of HIV transmission is based upon estimated differences in HIV transmission according to physiology (e.g. Boily et al., 2009). Therefore, where model results are presented alone, the results are discussed in regard to physiological "sex". Where model results are presented in concert with observed results, for instance where trial questionnaires may have asked about a participant's gender, results are presented according to "sex/gender".

References

Beaumont (2010) Approximate Bayesian Computation in Evolution and Ecology. Annual Review of Ecology, Evolution, and Systematics. 41. 379-406. doi:10.1146/annurev-ecolsys-102209-144621

Boily MC, Baggaley RF, Wang L, Masse B, White RG, Hayes RJ, Alary M. Heterosexual risk of HIV-1 infection per sexual act: systematic review and meta-analysis of observational studies. Lancet Infect Dis. 2009 Feb;9(2):118-29. doi: 10.1016/S1473-3099(09)70021-0. PMID: 19179227; PMCID: PMC4467783.

Central Statistical Office/Zambia, Central Board of Health/Zambia, and ORC Macro. 2003. Zambia Demographic and Health Survey 2001-2002. Calverton, Maryland, USA: Central Statistical Office/Zambia, Central Board of Health/Zambia, and ORC Macro.

Central Statistical Office/Zambia, Ministry of Health/Zambia, Tropical Diseases Research Centre/Zambia, University of Zambia, and Macro International. 2009. Zambia Demographic and Health Survey 2007. Calverton, Maryland, USA: Central Statistical Office/Zambia and Macro International Inc.

Central Statistical Office/Zambia, Ministry of Health/Zambia, and ICF International. 2014. Zambia Demographic and Health Survey 2013-14. Rockville, Maryland, USA: Central Statistical Office/Zambia, Ministry of Health/Zambia, and ICF International.

Donnell D, Floyd S, Hayes R (2018) Population Effects of Antiretroviral Therapy to Reduce HIV Transmission (PopART): A cluster-randomized trial of the impact of a combination prevention package on population-level HIV incidence in Zambia and South Africa. Statistical Analysis Plan. v3.0 16 December 2018. URL: https://www.hptn.org/sites/default/files/2019-01/HPTN071_SAP_v3.0_16Dec2018.pdf

Easterbrook PJ, Farzadegan H, Hoover DR, Palenicek J, Chmiel JS, Kaslow RA, Saah AJ. Racial differences in rate of CD4 decline in HIV-1-infected homosexual men. AIDS. 1996 Sep;10(10):1147-55. PMID: 8874633.

Ellenberg et al., (2007) Data Monitoring Committees in Clinical Trials: A Practical Perspective. Statistics in Practice. Wiley, England.

Floyd S, Ayles H, Schaap A, Shanaube K, MacLeod D, Phiri M, et al. (2018) Towards 90-90: Findings after two years of the HPTN 071 (PopART) cluster-randomized trial of a universal testing-and-treatment intervention in Zambia. PLoS ONE 13(8): e0197904. https://doi.org/10.1371/journal.pone.0197904

Floyd S, Shanaube K, Yang B, Schaap A, Griffith S, Phiri M, et al. (2020) HIV testing and treatment coverage achieved after 4 years across 14 urban and peri-urban communities in Zambia and South Africa: An analysis of findings from the HPTN 071 (PopART) trial. PLoS Med 17(4): e1003067. https://doi.org/10.1371/journal.pmed.1003067

Gona, P.N., Gona, C.M., Ballout, S. *et al.* Burden and changes in HIV/AIDS morbidity and mortality in Southern Africa Development Community Countries, 1990–2017. *BMC Public Health* **20,** 867 (2020). https://doi.org/10.1186/s12889-020-08988-9

Granich R, Kahn JG, Bennett R, Holmes CB, Garg N, Serenata C, et al. (2012) Expanding ART for Treatment and Prevention of HIV in South Africa: Estimated Cost and Cost-Effectiveness 2011-2050. PLoS ONE 7(2): e30216. https://doi.org/10.1371/journal.pone.0030216

Hayes RJ and Moulton LH (2022) Cluster Randomised Trials, 2nd Ed. Chapman and Hall/CRC.

Hayes R, Sabapathy K, Fidler S. Universal testing and treatment as an HIV prevention strategy: research questions and methods. Curr HIV Res. 2011;9(6):429-445. doi:10.2174/157016211798038515

Herbeck J, Tanser F. Community viral load as an index of HIV transmission potential. Lancet HIV. 2016 Apr;3(4):e152-4. doi: 10.1016/S2352-3018(16)00036-9

HPTN 071 (PopART) Trial Protocol V3.0 (2015). URL: https://www.hptn.org/sites/default/files/inline-files/HPTN%20Protocol%20071%20V.3.0-%2016%20Nov%202015%20Final.pdf. Date accessed: 3 July 2021.

Human Sciences Research Council (HSRC). South African HIV/AIDS, Behavioural Risks, Sero-status, and Mass Media Impact Survey (SABSSM) 2002: Adult and youth data - All provinces. [Data set]. SABSSM 2002 Adult-youth. Version 1.0. Pretoria South Africa: Human Sciences Research Council [producer] 2002, Human Sciences Research Council [distributor] 2011. http://dx.doi.org/doi:10.14749/1400830395.

Human Sciences Research Council (HSRC). *South African National HIV Prevalence, HIV Incidence, Behaviour and Communication Survey (SABSSM) 2005: Adult and youth data - All provinces*. [Data set]. SABSSM 2005 Adult-youth. Version 1.0. Pretoria South Africa: Human Sciences Research Council [producer] 2005, Human Sciences Research Council [distributor] 2011. http://dx.doi.org/doi:10.14749/1400830455.

Human Sciences Research Council (HSRC). *South African National HIV Prevalence, HIV Incidence, Behaviour and Communication Survey (SABSSM) 2008a: Adult - All provinces*. [Data set]. SABSSM 2008 Adult. Version 1.0. Pretoria South Africa: Human Sciences Research Council [producer] 2009, Human Sciences Research Council [distributor] 2014. http://dx.doi.org/doi:10.14749/1434098373.

Human Sciences Research Council (HSRC). *South African National HIV Prevalence, HIV Incidence, Behaviour and Communication Survey (SABSSM) 2008b: Youth - All provinces*. [Data set]. SABSSM 2008 Youth. Version 1.0. Pretoria South Africa: Human Sciences Research Council [producer] 2009, Human Sciences Research Council [distributor] 2014. http://dx.doi.org/doi:10.14749/1412873886.

Human Sciences Research Council (HSRC). *South African National HIV Prevalence, HIV Incidence, Behaviour and Communication Survey (SABSSM) 2012: Combined - All provinces*. [Data set]. SABSSM 2012 Combined. Version 2.0. Pretoria South Africa: Human Sciences

Research Council [producer] 2012, Human Sciences Research Council [distributor] 2018. http://dx.doi.org/doi:10.14749/1517402043.

Klein MB, Young J, Dunn D, Ledergerber B, Sabin C, Cozzi-Lepri A, Dabis F, Harrigan R, Tan DH, Walmsley S, Gill J, Cooper C, Scherrer AU, Mocroft A, Hogg RS, Smaill F; Canadian−European Clade Collaboration. The effects of HIV-1 subtype and ethnicity on the rate of CD4 cell count decline in patients naive to antiretroviral therapy: a Canadian-European collaborative retrospective cohort study. CMAJ Open. 2014 Oct 1;2(4):E318-29. doi: 10.9778/cmajo.20140017. PMID: 25485259; PMCID: PMC4251518.

Lash T et al., (2021) Modern Epidemiology. 4th Ed. LWW.

Lenormand, M., Jabot, F. and Deffuant, G. (2011). Adaptive Approximate Bayesian Computation for complex models. arXiv:1111.1308.

Mangal, Tara D. on behalf of the UNAIDS Working Group on CD4 Progression and Mortality Amongst HIV Seroconverters including the CASCADE Collaboration in EuroCoord Joint estimation of CD4+ cell progression and survival in untreated individuals with HIV-1 infection, AIDS: May 15, 2017 - Volume 31 - Issue 8 - p 1073-1082. doi: 10.1097/QAD.0000000000001437

McKinley, T. J., Vernon, I., Andrianakis, I., McCreesh, N., Oakley, J. E., Nsubuga, R. N., Goldstein, M. and White, R. G. (2018). Approximate Bayesian Computation and simulation-based inference for complex stochastic epidemic models. Statist. Sci. 33 4–18. URL: https://doi.org/10.1214/17-STS618

Montaner JS, Hogg R, Wood E, Kerr T, Tyndall M, Levy AR, Harrigan PR. The case for expanding access to highly active antiretroviral therapy to curb the growth of the HIV epidemic. Lancet. 2006 Aug 5;368(9534):531-6. doi: 10.1016/S0140-6736(06)69162-9. PMID: 16890841.

Müller V, von Wyl V, Yerly S, Böni J, Klimkait T, Bürgisser P, Ledergerber B, Günthard HF, Bonhoeffer S; Swiss HIV Cohort Study. African descent is associated with slower CD4 cell count decline in treatment-naive patients of the Swiss HIV Cohort Study. AIDS. 2009 Jun 19;23(10):1269-76. doi: 10.1097/QAD.0b013e32832d4096. PMID: 19461503.

Pickles M, Cori A, Probert WJM, Sauter R, Hinch R, Fidler S, et al. (2021) PopART-IBM, a highly efficient stochastic individual-based simulation model of generalised HIV epidemics developed in the context of the HPTN 071 (PopART) trial. PLoS Comput Biol 17(9): e1009301. https://doi.org/10.1371/journal.pcbi.1009301

Probert, Sauter et al., (2018) Analysis Plan for Individual-based Model Projections of the HPTN 071 (PopART) Intervention. Version 1.0. December 14, 2018. URL: https://www.hptn.org/sites/default/files/2018-12/HPTN071_PopART_AnalysisPlan_IBMprojections.pdf

Rue H, Martino S, and Chopin N. Approximate Bayesian inference for latent Gaussian models using integrated nested Laplace approximations (with discussion). Journal of the Royal Statistical Society, Series B, 71(2):319{392, 2009.

Silverberg MJ, Leyden W, Quesenberry CP Jr, Horberg MA. Race/ethnicity and risk of AIDS and death among HIV-infected patients with access to care. J Gen Intern Med. 2009;24(9):1065-1072. doi:10.1007/s11606-009-1049-y

Simpson, D. P., H. Rue, A. Riebler, T. G. Martins, and S. H. Sørbye. 2017. “Penalising Model Component Complexity: A Principled, Practical Approach to Constructing Priors.” *Statistical Science* 32 (1): 1–28.

Solomon, Sunil Suhas et al. (2016) Community viral load, antiretroviral therapy coverage, and HIV incidence in India: a cross-sectional, comparative study. The Lancet HIV, Volume 3, Issue 4, e183 - e190

WHO (2020) Consolidated HIV strategic information guidelines: driving impact through programme monitoring and management. Geneva: World Health Organization; 2020. Licence: CC BY-NC-SA 3.0 IGO. URL: https://www.who.int/publications/i/item/9789240000735. Date accessed: 18 September 2021.

HPTN 071 study team

We are grateful to all members of the HPTN 071 (PopART) Study Team, and to the study participants and their communities, for their contributions to the research.
The HPTN 071 (PopART) Study Team: Richard Hayes (London School of Hygiene & Tropical Medicine, UK), Sarah Fidler (Imperial College, UK), Nulda Beyers (University of Stellenbosch, South Africa), Helen Ayles (Zambart, Zambia; and London School of Hygiene & Tropical Medicine, UK), Peter Bock (University of Stellenbosch, South Africa), Wafaa El-Sadr (HIV Prevention Trials Network [HPTN] Leadership and Operations Centre, USA), Myron Cohen (HPTN Leadership and Operations Centre, USA), Susan Eshleman (HPTN Laboratory Centre, Johns Hopkins University, USA), Yaw Agyei (HPTN Laboratory Centre, Johns Hopkins University, USA), Estelle Piwowar-Manning (HPTN Laboratory Centre, Johns Hopkins University, USA), Virginia Bond (Zambart, Zambia; and London School of Hygiene & Tropical Medicine, UK), Graeme Hoddinott (University of Stellenbosch, South Africa), Deborah Donnell (HPTN Statistical and Data Management Centre [SDMC], USA), Sian Floyd (London School of Hygiene & Tropical Medicine, UK), Ethan Wilson (HPTN SDMC, USA), Lynda Emel (HPTN SDMC, USA), Heather Noble (HPTN SDMC, USA), David Macleod (London School of Hygiene & Tropical Medicine, UK), David Burns (NIAID, USA), Christophe Fraser (University of Oxford, UK), Anne Cori (Imperial College, UK), Nirupama Sista (HPTN Leadership and Operations Centre, USA), Sam Griffith (HPTN Leadership and Operations Centre, USA), Ayana Moore (HPTN Leadership and Operations Centre, USA), Tanette Headen (HPTN Leadership and Operations Centre, USA), Rhonda White (HPTN Leadership and Operations Centre, USA), Eric Miller (HPTN Leadership and Operations Centre, USA), James Hargreaves (London School of Hygiene & Tropical Medicine, UK), Katharina Hauck (Imperial College, UK), Ranjeeta Thomas (Imperial College, UK), Mohammed Limbada (Zambart, Zambia), Justin Bwalya (Zambart, Zambia), Michael Pickles (Imperial College, UK), Kalpana Sabapathy (London School of Hygiene & Tropical Medicine, UK), Ab Schaap (Zambart, Zambia; and London School of Hygiene & Tropical Medicine, UK), Rory Dunbar (University of Stellenbosch, South Africa), Kwame Shanaube (Zambart, Zambia), Blia Yang (University of Stellenbosch, South Africa), Musonda Simwinga (Zambart, Zambia), Peter Smith (Imperial College, UK), Sten Vermund (HPTN Executive Committee), Nomtha Mandla (University of Stellenbosch, South Africa), Nozizwe Makola (University of Stellenbosch, South Africa), Anneen van Deventer (University of Stellenbosch, South Africa), Anelet James (University of Stellenbosch, South Africa), Karen Jennings (City Health Department, Cape Town, South Africa), James Kruger (Department of Health, Western

Cape), Mwelwa Phiri (Zambart, Zambia), Barry Kosloff (Zambart, Zambia; and London School of Hygiene & Tropical Medicine, UK), Lawrence Mwenge (Zambart, Zambia), Sarah Kanema (Zambart, Zambia), Rafael Sauter (University of Oxford, UK), William Probert (University of Oxford, UK), Ramya Kumar (Zambart, Zambia; and London School of Hygiene & Tropical Medicine, UK), Ephraim Sakala (Zambart, Zambia), Andrew Silumesi (Ministry of Health, Zambia), Tim Skalland (HPTN SDMC, USA), Krista Yuhas (HPTN SDMC, USA).

HPTN 071 (PopART) Data Sharing policy

Background

NIAID, HPTN, LSHTM, and other PopART partners have general expectations and/or policies to make research data available to other researchers in a collaborative manner. However, there is need for an agreed, comprehensive and detailed data sharing policy specific to this study to be widely shared so that all stakeholders share the same expectations for when and what data will be made available, and through what mechanisms. Such a policy will ensure that HPTN 071 protocol team members and stakeholders have a period of priority access to the data, while still providing a pathway for more public access over time, and will strive to ensure that public use represents the data responsibly. As well, a comprehensive policy will avoid reliance on multiple "one-off" data sharing policies or mechanisms that might otherwise arise and be inconsistent with one another. It will enable the team to address expectations and requirements of funding agencies, journals, conferences and the broader scientific community, and will maximise the public policy and health benefit that may be derived from the data.

Types of HPTN 071 (PopART) Data Addressed by this Policy

There are three main types of HPTN 071 study data:

1. Data obtained from the Population Cohort (PC)
    a. Survey data collected by PC field teams
    b. Data obtained from analysis of blood samples obtained from PC participants during household visits by the PC field teams
2. Data obtained by intervention field teams
    a. Data obtained during household visits in Arm A & B communities.

Intervention data available aggregated by sex, age group and community.

3. Data regarding stigma
    a. Quantitative data regarding HIV stigma obtained from health workers in the communities.

Principles forming the basis of the HPTN 071 (PopART) data sharing policy

- The HPTN 071 (PopART) protocol team agrees that wider access to research data is in the public interest.

- Secondary analyses arising as a result of data sharing should meet the same high standards expected of the HPTN and HPTN 071 (PopART) analyses.

- The HPTN is committed to building scientific capacity among early career investigators at the research sites in Africa, and this requires that the study team should have exclusive access to the data for a defined period.

- Data arising from research with human subjects have special ethical considerations in terms of confidentiality and consent.

- Because the HPTN 071 (PopART) study is an interventional trial designed to answer a specific set of research questions, the data may not always be appropriate to answer other questions.

- Conclusions derived from misunderstandings or erroneous analyses of data can harm the reputation of a study, destroy the trust of the community, and discourage further participation in research. Therefore, the HPTN 071 (PopART) protocol team has a responsibility to ensure that data are accessed only by legitimate investigators who have agreed to abide by the provisions of this policy.

Key aspects of the HPTN 071 (PopART) data sharing policy

- Researchers from the HPTN 071 (PopART) study team who collected data have a legitimate interest in benefiting from their investment of time and effort, as well as a commitment to supporting capacity building for early career investigators at the study

sites. Therefore, the study team will have a period of *exclusive use* before the data are made available for sharing.

1. Exclusive use will be for a fixed period of 1 year after the publication of the primary results in the New England Journal of Medicine on 18 July 2019.
2. De-identified analysis datasets for the primary publication will be released to 3ie following publication of the primary manuscript, for replication purposes. Analysis datasets supporting other manuscripts will be posted as required by journals at the time of publication.
3. Data may be made available to researchers external to the study team earlier, with specific permission of the Protocol Chairs and Protocol Statistician when this does not conflict with the publication plans for the study.

- During and after the period of exclusive protocol team use, protocol team members with approved publication concepts are provided access to HPTN 071 PopART data stored at the HPTN SDMC by submission of a signed HPTN 071 Data Access Agreement. Protocol team members agree that they will only use the data for the analyses in the approved publication concept.

- During and after the period of exclusive protocol team use, protocol team members may use the data to pursue analyses agreed upon by protocol team leadership but not explicitly described in an approved publication concept, especially those requested by study funders or policymakers.

- After the period of exclusive use, data will be made available to users outside of the protocol team after an application and approval process (controlled public access).

- Requests for data are made in writing to the HPTN 071 (PopART) Publications Working Group, using a standard Data Access Proposal Form. Proposals are reviewed by the Publications Working Group, and access to study data is facilitated by the HPTN Statistical and Data Management Center (SDMC).

- Researchers external to the study protocol team who are granted access to data are encouraged to engage with the HPTN 071 (PopART) study team to ensure they have sufficient understanding of the study and the data elements.

- Any publications arising from the shared data must acknowledge the investigators who collected the data, the institutions involved, and the funding sources. A standard acknowledgement statement will be provided.

Storage for data sharing

- Data will be stored securely at the HPTN SDMC according to HPTN and HPTN 071 (PopART) Standard Operating Procedures (SOPs) and access provided to approved applicants.

- Authorized users will be granted access to all study data relevant to addressing the research question posed.

Standards for data sharing

Data cannot be used effectively unless they are thoroughly documented and their collection methods understood. Therefore, in general, data are made available for sharing when they are cleaned, documented and the appropriate metadata are in place. Documentation includes detailed descriptions of how the data were collected and coded, and how lab results were obtained. The annotated questionnaire will also be included. Data available for sharing will be from the final 'locked' database.

The following standards will be observed:

- Data will be de-identified before release for sharing, with all direct personal identifiers removed. Where there are indirect identifiers that could lead to deductive disclosure (e.g. GPS coordinates for a person's house), these will be modified or removed from the dataset. Any requests for access to a dataset that includes identifiers will need to be negotiated with the HPTN 071 (PopART) Publications Working Group. If necessary, such requests may need to be referred to relevant research and ethics committees.

- Data may be in separate files, but each file will have a data dictionary that identifies the key fields needed to merge files

- Data documentation will also include the names and institutions of the PIs who collected the data and details of the funding source of the study

Conditions of access for non-protocol team members

All requests to share data must be made in writing using the HPTN 071 (PopART) Data Access Application Form. Where relevant, an accompanying proposal detailing the study questions and analysis methods should be submitted as an appendix to the application form. Applications will be reviewed by the HPTN 071 Publications Working Group.

In order for access to be considered, the applicant must meet the following requirements:

- provide evidence of being a bona fide researcher in a field relevant to the study or on associated statistical methods (e.g. relevant peer-reviewed research that can be found on PubMed, or a successful application to a relevant, independent data access committee). If the primary investigator who will be using the data does not meet these criteria, they must identify a mentor who will take responsibility for supervising them and guaranteeing their compliance with this policy
- have an experienced statistician on their research team
- be willing to sign a data access agreement
- agree to notify the HPTN SDMC if any errors are identified in the data
- agree not to share the data with third parties without permission from the HPTN 071 Publications Working Group
- agree to share the methods and results of all the analyses using HPTN 071 (PopART) data with the HPTN 071 protocol chairs and protocol statistician when completed, and certainly no later than when analyses have been accepted for publication or presentation.

Priority will be given to applicants whose proposals include a plan for sharing analysis skills with site researchers. Access decisions will take into consideration the planned publications by the HPTN 071 (PopART) protocol team. The committee will make its decision within 4 weeks of the application being received. The aim will be that the data will then be transferred within 4 weeks after a positive decision. Where this is not possible, the Protocol Statistician at the HPTN SDMC will inform the HPTN 071 (PopART) Publications Working Group and the applicant in writing and will negotiate a later date.

Any publications arising from the shared data must acknowledge the research team who collected the data, the institutions involved, and funding sources, and must state

that HPTN 071 (PopART) investigators were not involved in the analysis (except in the case where there are protocol team collaborators). This acknowledgement statement will be provided to investigators with whom HPTN 071 (PopART) data are shared.
